# In vitro comparison of major memory-support dietary supplements for their effectiveness in reduction/inhibition of beta-amyloid protein fibrils and tau protein tangles: key primary targets for memory loss

**DOI:** 10.1038/s41598-020-79275-1

**Published:** 2021-02-15

**Authors:** Alan D. Snow, Joel A. Cummings, Rudolph E. Tanzi, Thomas Lake

**Affiliations:** 1 Cognitive Clarity Inc., Edmonds, WA 98026 USA; 2 Genetics and Aging Research Unit, Department of Neurology, Massachusetts General Hospital and Harvard Medical School, Charlestown, MA USA

**Keywords:** Alzheimer's disease, Acute inflammation, Cognitive ageing, Cognitive neuroscience, Diseases of the nervous system, Learning and memory

## Abstract

Memory loss is primarily caused by the accumulation of both brain plaques [(consisting of beta-amyloid protein (Aβ) 1–42)] and neurofibrillary tangles (consisting of paired helical and straight filaments containing tau protein). Neuroinflammation is the third key and important factor that leads to accelerated memory loss and eventual dementia. Brain plaques, tangles and inflammation is the trilogy mainly responsible for causing memory loss that has now been documented for over 20 years in the scientific literature. The present investigation used in vitro quantitative methods to directly compare the ability of major memory-support dietary supplements to reduce pre-formed Aβ 1–42 fibrils (21 supplements tested) and tau protein paired helical/straight filaments (13 supplements tested)—two of the three most important targets for memory loss. Additionally, 18 different manufacturers of cat’s claw (*Uncaria tomentosa*) were directly compared for their ability to inhibit/reduce Aβ 1–42 fibrils and/or tau paired helical/straight filaments based on recent findings that PTI-00703 cat’s claw is a specific and potent inhibitor/reducer of all three targets -brain plaques, tangles and inflammation (Snow et al. in Sci Rep 9:561, 2019). In the present investigation quantitative Thioflavin T fluorometry was used on a comparative weight-to-weight basis at increasing concentrations with ingredients tested from the actual capsules the consumer ingests. Major memory-support dietary supplements were directly compared for their ability to inhibit and disaggregate/reduce both Aβ 1–42 fibrils and/or tau paired helical/straight filaments. Dietary supplements touted to enhance memory comparatively tested included Prevagen, FOCUSfactor, PROCERA AVH, Alpha Brain, NAD^+^OVIM, BRAIN JUICE, Cebria, EXCELEROL, NOOCUBE, US Doctor’s Clinical Brain Power ADVANCED, healthycell pro, LUMONOL, Brain Awake, BRAIN ARMOR, brainMD (BRAIN & MEMORY POWER BOOST), Brain Support, Clarity (BRAIN HEALTH FORMULA), brainMD (NEUROVITE PLUS), neuriva (Original and Plus) and percepta. This is the first paper to actually comparatively test these memory-support supplements for their ability to reduce Aβ fibrils and tau protein tangles. Percepta (PTI-00703 cat’s claw and a specific oolong tea extract) was determined to be the most effective and potent memory support dietary supplement to disaggregate/disrupt Aβ 1–42 fibrils (range of 25–89%) and tau paired helical/straight filaments (range of 26–86%) at all 3–4 doses tested in comparison to other major memory-support dietary supplements tested. This was at least more than double (> 50%) for percepta reducing Aβ 1–42 fibrils and in comparison to the other 20 memory-support dietary supplements tested. The ranking order for memory-support supplement effects based on reducing Aβ 1–42 fibrils (Aβ 1–42: memory-support supplement at 1:0.1 weight-to-weight in a 3-day study) was percepta (69.6% reduction) >>> Alpha Brain (34.9% reduction) = US Doctor’s Clinical Brain Power ADVANCED (32.4%) = BRAIN JUICE (30.1%) = neuriva Plus (27%) = neuriva Original (27%) > NEUROVITE PLUS (22.9%) = NOOCUBE (19.9%) = EXCELEROL (17.3%) = healthycell pro (17.2%) > Prevagen (12.9%) > PROCERA AVH (6.5%) = FOCUSfactor (5.5%) > Cebria (0%) = Brain Awake (0%) = Brain Support (0%) = brainMD (BRAIN & MEMORY POWER BOOST) (0%) = NAD^+^OVIM (0%) = BRAIN ARMOR (0%) = LUMONOL (0%). The ranking order for memory support supplement effects on reducing tau paired helical/straight filaments (tau:memory supplement at 1:1 weight-to-weight at 3 days) was percepta (85.7% reduction) >>> neuriva Plus (57.9%) >> BRAIN JUICE (41.9%) = EXCELEROL (41.0%) = neuriva Original (38.4%) = US Doctor’s Clinical Brain Power ADVANCED (38.3%) = healthycell pro (37.6%) >> Alpha Brain (27.9%) >> NOOCUBE (17.6%) >> FOCUSfactor (8.7%) > Cebria (3.6%) = PROCERA AVH (0%) = Prevagen (0%). Congo red staining, Thioflavin S fluorescence, circular dichroism (CD) spectroscopy and electron microscopy confirmed the positive results observed with the supplement percepta. CD spectroscopy demonstrated that percepta caused a marked inhibition of beta-sheet secondary folding of tau protein into paired helical filaments. PTI-00703 cat’s claw (main ingredient in percepta) was also identified as the most potent cat’s claw bark powder (*Uncaria tomentosa*) to reduce and inhibit Aβ 1–42 fibrils and tau tangles in comparison to 17 other manufacturers of cat’s claw extracts. Although there are thousands of brain memory-support dietary supplements in the marketplace today, none of them have been directly compared and analyzed for their ability to reduce and/or inhibit two major targets of memory loss i.e. Aβ 1–42 fibrils and tau paired helical/straight filaments (major constituents of brain plaques and tangles). In our comparison studies, we show that percepta has the most potent ability to disaggregate/reduce Aβ 1–42 fibrils and tau protein paired helical/straight filaments as demonstrated by a variety of methods most likely due to the specific polyphenol content in PTI-00703 cat’s claw (i.e. polyphenols and proanthocyanidins) as we have previously shown (Snow et al. in Sci Rep 9:561, 2019). Memory-support dietary supplements tested that also contained polyphenols and/or cat’s claw in their product demonstrated some Aβ fibril and tau protein tangle reducing activity, but were much less effective than percepta. Percepta’s main ingredient, PTI-00703 cat’s claw, has previously been shown to reduce brain amyloid plaques and Aβ 1–42/40 insoluble/soluble levels in brain (in plaque-producing transgenic mice) with marked concurrent memory improvements (shown by Morris water maze testing) (Snow et al. in Sci Rep 9:561, 2019). The present investigation further confirms that percepta is one of the best dietary supplements that causes a marked reduction and inhibition of Aβ fibrils and tau tangle filaments -two important major targets for memory-support. In addition, PTI-00703 cat’s claw was the most effective cat’s claw (*Uncaria tomentosa*) ingredient for reducing /disaggregating and inhibiting Aβ 1–42 fibrils and tau protein paired helical/straight filaments in comparison to 17 other manufacturers of cat’s claw extracts tested.

## Introduction

Memory loss has been shown to be primarily caused by the accumulation of three major factors: (1) the accumulation and persistence of Swedish meatball looking-like amyloid plaques in brain between neurons consisting mainly of a 40–42 amino acid peptide called beta-amyloid protein or Aβ^1,2^, (2) the accumulation of dried-spaghetti-like strands inside dying neurons consisting primarily of a microtubule-associated protein called tau protein^3^; and (3) neuroinflammation^4^. The trilogy of memory loss leading to dementia and Alzheimer’s disease (AD) in nearly 100% of the cases is therefore the accumulation and persistence in brain of **P**laques, **T**angles and **I**nflammation (referred to as PTI)^5^.

As one normally ages, Aβ amyloid plaque accumulation occurs as early as 20 years old and increases progressively^6^. The build-up of tau protein in neurofibrillary tangles also increases during normal aging and is believed to occur shortly after brain plaque accumulation^7^. Normal brain aging in healthy individuals therefore involves the brain build-up of both plaques and tangles. When the accumulation of brain plaques and tangles begins to be excessive, memory loss and cognitive decline worsen, and can first appear clinically as mild cognitive impairment (MCI). Increased brain persistence of plaques and tangles associated with neuroinflammation is the tipping point of marked neuronal loss that can eventually lead to the diagnosis of AD (based on memory testing, the ruling out of other diseases and using brain PET imaging techniques in live patients to confirm the diagnosis)^8,9^. In AD, there is an accumulation of thousands, to hundreds of thousands of plaques and tangles in specific brain regions including hippocampus and cortex. If plaques and tangles are the matches that light the brain on fire, then neuroinflammation ignites the neuron-killing forest fire^10–13^. Thus, the trilogy of brain plaques, tangles and inflammation is postulated to lead to a marked potential and rapid decline in memory and cognition in the aging population. Although there may be other potential causes of memory loss including oxidative stress^14^, reduced blood flow^15^, reduced synaptic integrity^16,17^, reduced plasticity processes^18,19^, the most documented studies that lead to memory loss and eventual dementia is the accumulation of brain plaques (i.e. Aβ fibrils), neurofibrillary tangles (i.e. tau protein filaments) and neuroinflammation as observed in AD -the most common cause of memory loss problems among older adults^20^.

In our previous study, Snow et al.^5^ identified an extract from the woody vine, *Uncaria tomentosa* (i.e. cat’s claw) that markedly reduced and inhibited both Aβ amyloid plaques and tau protein tangles. A specific cat’s claw from bark powder of a woody vine growing deep in the Peruvian Amazon rain forest referred to as PTI-00703 cat’s claw was used. This led to the development of a unique dietary supplement for memory-support (called percepta) that basically consisted of just two bioactive ingredients, PTI-00703 cat’s claw and a specific oolong tea extract. 10 years of research studies with 8 different institutions led to a landmark paper by Snow et al.^5^ in which newly discovered polyphenols (i.e. specific proanthocyanidins or epicatechin-dimers) in PTI-00703 cat’s claw entered the brain within two minutes of being in the blood. In plaque-producing double mutation TASD-41 transgenic mice, the main proanthocyanidin B2 in PTI-00703 cat’s claw reduced brain plaque load (in older animals) by 57% and improved short-term memory (assessed by Morris water maze testing) by 58% following a short 3-month treatment. Neuroinflammation (both astrocytosis and microgliosis) was also markedly decreased by 69–80% in the brains of these animals following 3-months of treatment^5^. Percepta was developed to be the first dietary and memory-support supplement to specifically target brain plaques and tangles.

In the present studies, quantitative Thioflavin T fluorometry^21–23^ determined the effects of different major memory-support dietary supplements to disaggregate/dissolve Aβ 1–42 fibrils (major constituent in brain amyloid plaques) and/or tau protein containing paired helical/straight filaments (major constituent in brain neurofibrillary tangles). Other methods to confirm effects of the most effective memory-supplement identified to reduce Aβ 1–42 fibrils and tau protein tangles (i.e. percepta) included Congo red staining (i.e. red/green birefringence under polarized light)^24^, Thioflavin S fluorescence^25^, and electron microscopy (to visualize plaque amyloid fibrils or tangle paired helical filaments)^26^. Circular dichroism (CD) spectroscopy also determined its effects to reduce β-sheet secondary folding^5^.

In the present investigation, we compared major memory-support dietary supplements to reduce and/or inhibit brain Aβ 1–42 fibrils and/or tau protein paired helical/straight filaments. Comparisons were made on a direct weight-to-weight (wt/wt) basis on the actual capsule ingredients used for oral consumption by consumers. Both disruption/disaggregation of pre-formed Aβ 1–42 fibrils (21 memory-support products tested) and pre-formed tau filament tangles (13 memory-support products tested) were specifically analyzed. Comparisons were made between Prevagen, FOCUSfactor, PROCERA AVH, Alpha Brain (contains cat’s claw), NAD^+^OVIM (contains cat’s claw), BRAIN JUICE, Cebria, EXCELEROL, NOOCUBE (contains cat’s claw), US Doctor’s Clinical Brain Power ADVANCED, healthycell pro , LUMINOL, Brain Awake, BRAIN ARMOR, brainMD (BRAIN & MEMORY POWER BOOST), Brain Support, Clarity (BRAIN HEALTH FORMULA), brainMD (NEUROVITE PLUS), neuriva (neuriva Original and neuriva Plus) and percepta. Percepta was found by far to be the best dietary supplement that was a potent disaggregator/disruptor of Aβ 1–42 fibrils and tau tangles in comparison to the other major memory-support dietary supplements.

Additionally, we compared 18 different manufacturers of cat’s claw (*Uncaria tomentosa)* for their ability to reduce Aβ 1–42 fibrils, inhibit Aβ 1–40 fibril formation, and/or reduce tau tangles. PTI-00703 cat’s claw (the main ingredient in Percepta) demonstrated the most robust activity for Aβ fibril and tau tangle reduction/inhibition compared to all the other cat’s claw extracts tested from different manufacturers. Cat’s claw (*Uncaria tomentosa*) is a known and potent reducer of neuroinflammation due to its ability to reduce the inflammatory cytokines TNF-alpha and interleukin-1^27–31^. This study demonstrated that one of the best dietary supplements targeting Aβ fibrils (major constituent of brain plaques) and tau paired helical/straight filament (major constituent in brian neurofibrillary tangles) was percepta.

## Results

### Percepta is the most effective reducer of brain Aβ 1–42 plaque fibrils in comparison to 20 other major memory-support dietary supplements

Thioflavin T fluorometry quantitated, differentiated and compared the effects of different major memory-support dietary supplements on Aβ 1–42 fibril reduction and disaggregation (Table 1). Aβ 1–42 protein is the major protein found in amyloid plaques in the aging brain and in AD^32^. In the first set of studies, 21 different memory-support dietary supplements were compared directly to each other on a weight-to-weight (wt/wt) basis for their ability to disaggregate and reduce pre-formed Aβ 1–42 fibrils. Brain supplements directly compared in different sets of studies were percepta, Prevagen, FOCUSfactor, PROCERA AVH, Alpha Brain (contains cat’s claw), NAD^+^OVIM (contains cat’s claw), BRAIN JUICE, Cebria, EXCELEROL, NOOCUBE (contains cat’s claw), US Doctor’s Clinical Brain Power ADVANCED, healthycell pro, LUMONOL, Brain Awake, BRAIN ARMOR, brainMD (BRAIN & MEMORY POWER BOOST), Brain Support, Clarity (BRAIN HEALTH FORMULA), brainMD (NEUROVITE PLUS), and neuriva (neuriva Original and neuriva Plus) (Table 1).

**Table 1:** Comparison of 21 memory-support supplements for reduction of Aβ 1–42 fibril and/or tau tangles.

Dietary supplement product name/company	Major ingredients/daily serving size (total)	% Reduction of Aβ 1–42 fibrils (1:0.1 wt/wt)	% Reductio of Tau Tangles (1:1 wt/wt)	Solubility issues	Reported mechanism of action/claims
Percepta (Cognitive Clarity Inc.)	PTI-00703 cat’s claw; oolong tea extract (2 capsules/750–775 mg)	69.6	85.7	No	Targets/reduces brain plaques and tangles; improves memory, focus and concentration
Alpha Brain (Onnit Labs)	l-tyrosine; l-theanine; PS; cat’s claw; GPC; bacopin; vinpocetine (2 capsules/1315 mg)	34.9	27.9	No	Improves memory, focus and processing speed
US Doctor’s Clinical Brain Power ADVANCED (US Doctors Clinical)	Vitamin B6; B12; ginkgo biloba; green tea; choline; huperzine A; vinpocetine; acetyl-l-carnitine (2 capsules/1086 mg)	32.4	38.3	No	Supports memory and mental sharpness; promotes healthy circulation in the brain
BRAIN JUICE (Brain Juice)	Vitamin C; B6; B12; choline; green tea; acetyl-l-carnitine; acai berry; blueberry; l-theanine (1 bottle/2.5 fl. oz.)	30.1	41.9	No; used liquid	Focus; Clarity; Memory; Mood
Neuriva Plus (Schiff Vitamins)	Coffee fruit extract; PS (1 capsule; ~ 302 mg)	27.0	57.0	No	Supports focus, memory, learning, accuracy and concentration
Neuriva Original (Schiff Vitamins)	Coffee fruit extract; PS (1 capsule; 200 mg)	27.0	38.4	No	Supports focus, memory, learning, accuracy and concentration
brainMD (NEUROVITE PLUS)	Vitamins; calcium; magnesium; zinc; choline; hesperidin; blueberries; strawberries; broccoli; spinach; PS; coenzyme Q10;	22.9	NT	No	Multiple nutrients for sharp minds and healthy bodies
NOOCUBE (Wolfsen Berg Ltd)	Alpha-GPC; cat’s claw; oat straw; huperzine A; bacopin; l-theanine; l-tyrosine; vinpocetine; pterostilbene (2 capsules/997.5 mg)	19.9	17.6	No	Improves your cognitive functioning
EXCELEROL (Accelerated Intelligence Inc.)	Vitamin B12; niacin; Huperzine A; vinpocetine; acetyl-l carnitine; Bacopin; green tea; ginkgo biloba; peppermint; PS; l-tyrosine; white tea; black tea (2 capsules/ ~ 325 mg)	17.3	41.0	No	Supports memory, concentration and alertness
Healthycell pro (HealthyCell)	Vitamins; Minerals; Bilberry; Rosemary; Pycnogenol; Lutein; cat’s claw; turmeric; milk thistle; Korean ginseng; coenzyme Q10; N-acetyl-cysteine; gotu kola; l-theanine; green tea; resveratrol; pterostilbene;	17.2	37.6	No	Supports energy and mental focus
Prevagen (Quincy Biosciences)	Apoaequorin (jelly fish protein) (1 capsule/10 mg)	12.9	0	Small dispersible particles; dissolvable	Improves memory
PROCERA AVH (Procera Health)	Acetyl-l-carnitine; vinpocetine; huperzine A (3 capsules/1515 mg)	6.5	0	No	Supports brain health and cognitive function
FOCUSfactor (Synergy CHC Corp.)	Various vitamins; bacopin; DHA; bilberry fruit extract; grape seed extract; huperzine A; (4 tablets/2768 mg)	5.5	8.7	No	Improves memory, concentration and focus
Clarity (Neovicta)	Vitamins; green tea; bacopin; bilberry; grape seed extract; PS; cinnamon bark; N-acetyl-tyrosine	5.1	NT	No	Improves focus and memory; higher mental processing; boost creativity
Cebria (Cebria LLC)	Vitamin B6; B12; folic acid; l-theanine; green tea; amino acids (1 capsule/459 mg)	0.0	3.6	No	Significantly improves memory & recall
Brain Awake (Irwin Naturals)	Vitamin B6; B12; MCT oil; bacopin; l-theanine; rosemary; lemon balm; black pepper; tea extract (3 soft gels/2218 mg)	0.0	NT	No	Promotes focus and mental clarity; support retention of information; enhance performance on cognitive tasks
Brain Support (1 Body)	Vitamin B6; B12; alpha GPC; lion’s mane; bacopin; tyrosine; ginkgo biloba; PS; l-theanine; taurine; huperzine A; (3 capsules/2,152 mg)	0.0	NT	No	Supports memory and cognition
BrainMD (BRAIN & MEMORY POWER BOOST) (BrainMD Health)	PS; acetyl-cysteine; ginkgo biloba; vinpocetine; huperzine A;	0.0	NT	No	Supports healthy brain and memory performance
NAD^+^OVIM (Innovative Medicine LLC)	Magnesium; thiamine; bacopin; cat’s claw; NAD + ; co-enzyme Q10 (2 capsules/911 mg)	0.0	NT	No	Supports brain function, focus and concentration
BRAIN ARMOR (Brain Armor Inc)	Omega-3′s; DHA; EPA; MCT oil (2 capsules; 2028 mg)	0.0	NT	No	For strong brains
LUMONOL (Advanse Nutraceuticals)	*N*-acetyl l-tyrosine; l-theanine; caffeine (2 capsules; 600 mg)	0.0	NT	No	Helps improve focus; pre-workout energy booster

Major ingredients and reported mechanism of action/claims are also compared for each product

*DHA* docosahexaenoic acid, *NT* not tested, *EPA* eicosapentaenoic acid, *MCT* medium chain triglycerides, *NAD+* nicotinamide adenine dinucleotide, *PS* phosphatidylserine.

**Figure 1:**
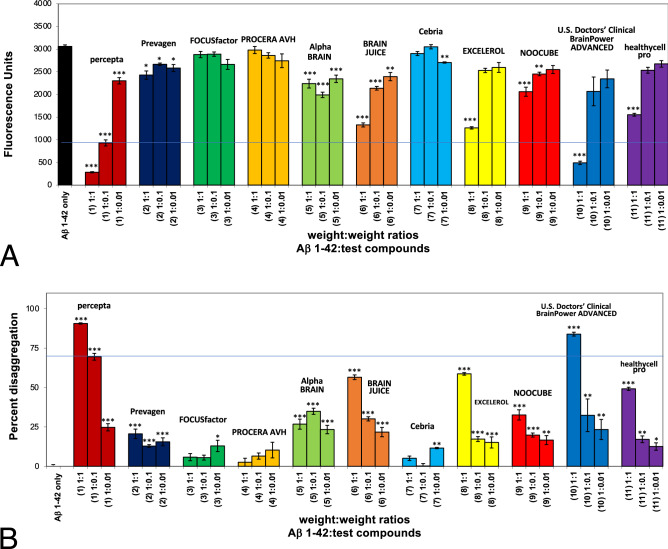
Percepta is the most potent disrupter of Aβ 1–42 fibrils in comparison to ten (10) other top-selling brain supplements. (**A**) Percepta (containing PTI-00703 cat’s claw and a specific oolong tea extract) was compared to Prevagen, FOCUSfactor, PROCERA AVH, Alpha Brain (contains cat’s claw), BRAIN JUICE, Cebria, EXCELEROL, NOOCUBE (contains cat’s claw), US Doctor’s Clinical Brain Power ADVANCED and healthycell pro (contains cat’s claw) for disruption of Aβ 1–42 fibrils. Percepta was the most potent disrupter /disaggregator of pre-formed Aβ 1–42 fibrils than any of the other 10 brain supplements tested (on a wt/wt basis) as quantitatively determined by Thioflavin T fluorometry. Aβ 1–42 formed fibrils instantly and showed 3,050 Thioflavin T fluorescent units (FU) due to immediate fibril formation (black bar). Increasing doses of percepta (Aβ 1–42:percepta wt/wt ratios of 1:0.01; 1:0.1; and 1:1) demonstrated a marked dose-dependent disaggregation/disruption of Aβ 1–42 fibrils. Amyloid plaque fibril reduction at 1:0.1 wt/wt is compared (blue line across graph). *p < 0.05, **p < 0.01, ***p < 0.001, by Student’s t-test. Bars represent mean ± SEM. (**B**) Comparison of percepta to 10 (ten) other top selling brain supplements in % disaggregation of preformed Aβ 1–42 fibrils. Percepta had the most robust activity and reduced/disaggregated Aβ 1–42 fibrils in a dose-dependent manner (light brown bars), better than other memory-support supplements including those that contained cat’s claw (i.e. Alpha Brain, NOOCUBE and healthycell pro). At a Aβ:percepta wt:wt ratio of 1:0.1 (blue line across graph), percepta disaggregated Aβ 1–42 fibrils by 69.6%, much better than any other brain supplement tested. *p < 0.05, **p < 0.01, ***p < 0.001, by Student’s t-test. Bars represent mean ± SEM.

As demonstrated in Fig. 1A, Aβ 1–42 was first incubated at 37 °C to observe instant amyloid fibril formation by Thioflavin T fluorometry at day 0 (3,050 fluorescent units; black bar on left). This was confirmed by positive Congo red staining (i.e. red-green birefringence as viewed under polarized light) (Fig. 5, top left)^5^ and positive Thioflavin S fluorescence (Fig. 5, middle left)^5^. Aβ 1–42 also demonstrated amyloid fibrils at day 0, as shown by negative stain electron microscopy (Fig. 5, lower left)^5^. In a first study (Fig. 1A), percepta was identified as the most potent disrupter of Aβ 1–42 fibrils in comparison to ten (10) other major memory-support dietary supplements on a wt/wt basis and in a dose-dependent manner (Aβ 1–42:dietary supplement ratios tested of 1:0.01; 1:0.1 and 1:1). Increasing doses of percepta demonstrated a marked dose-dependent disaggregation/disruption of pre-formed Aβ 1–42 fibrils (Fig. 1A,B). Aβ 1–42 amyloid plaque fibril reduction at the pre-determined clinical dose of percepta (i.e. 1:0.1 wt/wt)^5^ caused a significant (p < 0.001) 69.6% disaggregation/reduction of pre-formed Aβ 1–42 fibrils (Fig. 1B). Most of the other memory-support dietary supplements tested had much less effects on reduction of Aβ 1–42 fibrils at the 1:0.1 wt/wt dose (Table 1).

**Figure 2:**
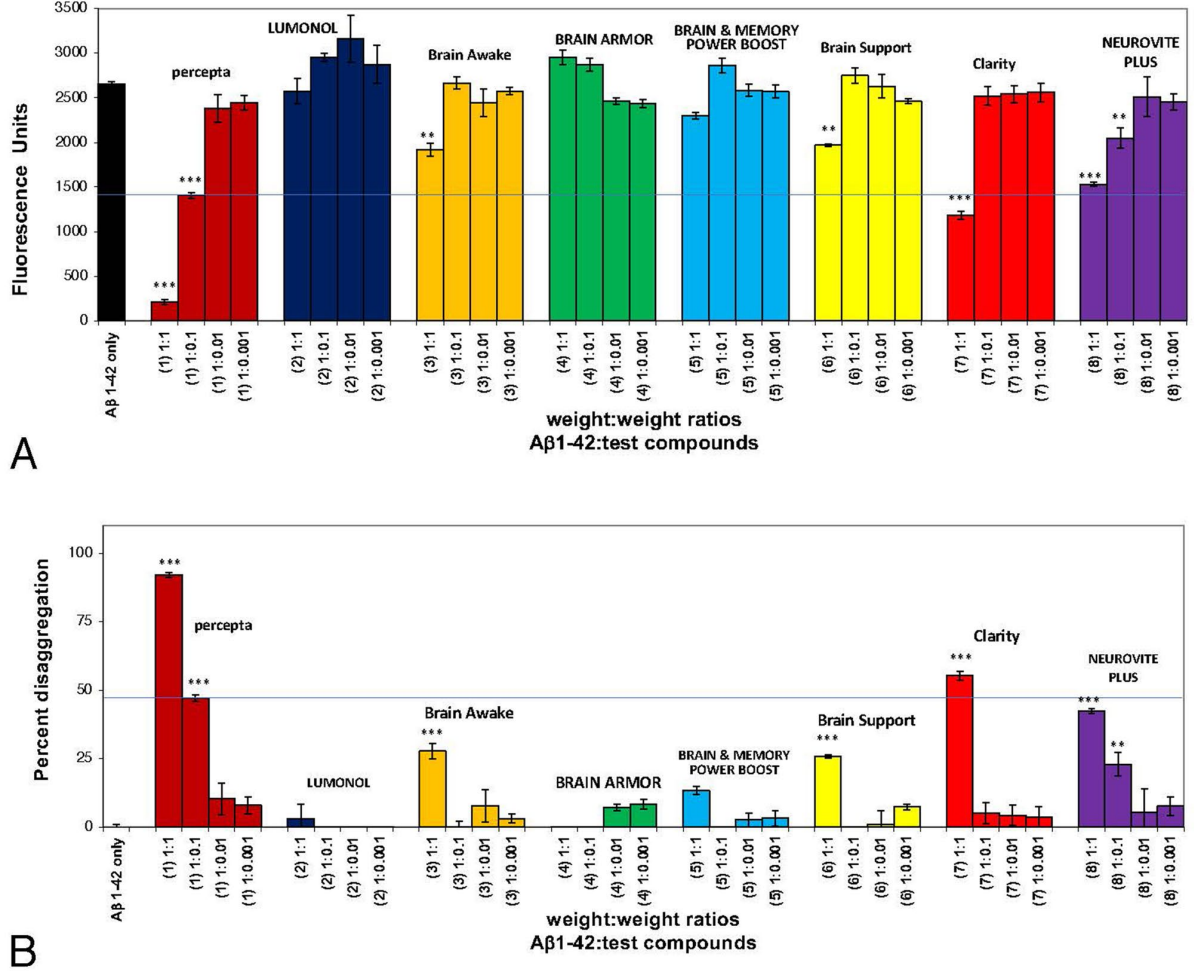
Percepta was the most potent disrupter of Aβ 1–42 fibrils in comparison to seven (7) other top-selling brain supplements on a wt/wt basis. (**A**) Percepta was compared to LUMONOL, Brain Awake, BRAIN ARMOR, brainMD (BRAIN & MEMORY POWER BOOST), Brain Support, Clarity (BRAIN HEALTH FORMULA), and brainMD (Neurovite Plus). Percepta, once again was the most potent disrupter /disaggregator of pre-formed Aβ 1–42 fibrils than any other brain supplement tested as determined quantitatively by Thioflavin T fluorometry. Aβ 1–42 formed fibrils instantly and showed 2,654 Thioflavin T fluorescent units (FU)(black bar). Increasing doses of percepta (Aβ 1–42:percepta wt/wt ratios of 1:0.001, 1:0.01; 1:0.1; and 1:1 were tested) demonstrated a marked dose-dependent disaggregation/disruption of pre-formed Aβ 1–42 fibrils. All the other brain supplements tested did not reduce Aβ 1–42 fibrils or only somewhat did so at the highest 1:1 wt/wt dose tested. *p < 0.05, **p < 0.01, ***p < 0.001, by Student’s t-test. Bars represent mean ± SEM. (**B**) Comparison of percepta to 10 (ten) other memory-support supplements in % disaggregation of preformed Aβ 1–42 fibrils. Percepta , by far, had the most robust activity and reduced/disaggregated Aβ 1–42 fibrils in a dose-dependent manner (light brown bars). At a Aβ:percepta wt:wt ratio of 1:0.1 (blue line across graph), percepta disaggregated Aβ 1–42 fibrils by 4.29%, much better than any other brain supplement tested (see line across the graph). *p < 0.05, **p < 0.01, ***p < 0.001, by Student’s t-test. Bars represent mean ± SEM.

As shown in Fig. 2A, in a second comparison study, Aβ 1–42 first incubated at 37 °C demonstrated instant amyloid fibril formation by Thioflavin T fluorometry at day O (i.e. 2654 fluorescence units; black bar on left). Percepta was again identified as the most potent disrupter of Aβ 1–42 amyloid plaque fibrils in comparison to seven (7) other memory-support dietary supplements on a wt/wt basis and in a dose-dependent manner (Aβ 1–42:dietary supplement ratios tested of 1:0.001, 1:0.01; 1:0.1 and 1:1) (Fig. 2A). Aβ 1–42 amyloid plaque fibril reduction at the pre-determined clinical dose of percepta (i.e. 1:0.1 wt/wt)^5^ caused a significant (p < 0.001) 43.9% disaggregation/reduction of pre-formed Aβ 1–42 fibrils (Fig. 2B). None of the other brain supplements were even close to reducing Aβ 1–42 fibrils in comparison to percepta on a wt/wt basis (Table 1).

**Figure 3:**
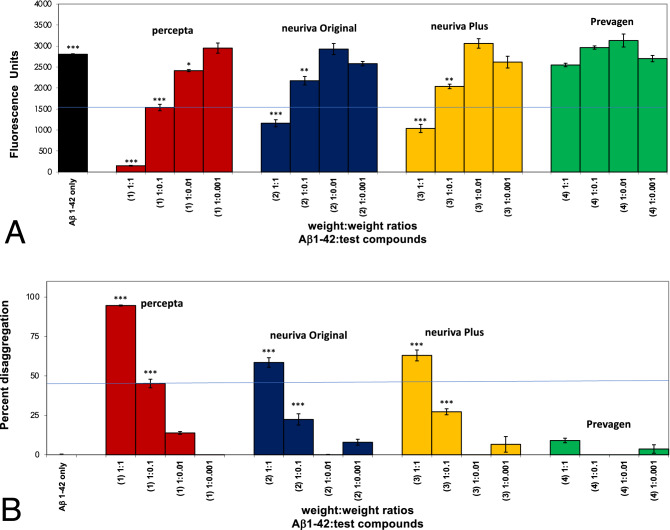
Percepta outperformed neuriva (Schiff Vitamins) for disaggregation/disruption of pre-formed Aβ 1–42 fibrils. (**A**) Percepta was tested in comparison to neuriva (neuriva Original and neuriva Plus) and Prevagen memory-support supplements on a wt/wt basis. Percepta was the most potent disrupter of Aβ 1–42 fibrils in comparison to neuriva (neuriva Original and neuriva Plus) and Prevagen on a wt/wt basis as quantitatively determined by Thioflavin T fluorometry. Aβ 1–42 fibrils demonstrate binding to Thioflavin T (indicator of fibril formation) and 2800 fluorescence units. Percepta demonstrated the most robust disaggregation/dissolution of pre-formed Aβ 1–42 fibrils. *p < 0.05, **p < 0.01, ***p < 0.001, by Student’s t-test. Bars represent mean ± SEM. (**B**) Comparison (on a wt/wt basis) of percepta to neuriva (Original and Plus) and Prevagen for % disaggregation of preformed Aβ 1–42 fibrils at 4 increasing dosages. Percepta had the most robust activity and reduced/disaggregated Aβ 1–42 fibrils in a dose-dependent manner (light brown bars). At a Aβ:percepta wt:wt ratio of 1:0.1 (blue line across graph), percepta disaggregated Aβ 1–42 fibrils by 45.3%, whereas neuriva Original at that dose only disaggregated Aβ 1–42 fibrils by 25.4%, neuriva Plus only disaggregated Aβ 1–42 fibrils by 30.2%, and Prevagen did not work on amyloid plaque fibrils at all. *p < 0.05, **p < 0.01, ***p < 0.001, by Student’s t-test. Bars represent mean ± SEM.

As shown in Fig. 3A and B, in a third comparison study, Aβ 1–42 first incubated at 37 °C demonstrated instant amyloid fibril formation by Thioflavin T fluorometry at day O (i.e. 2800 fluorescence units; black bar on left). Percepta was again identified as the most potent disrupter of Aβ 1–42 fibrils in comparison to neuriva Original, neuriva Plus and Prevagen on a wt/wt basis and in a dose-dependent manner (Aβ 1–42:dietary supplement ratios tested of 1:0.001, 1:0.01; 1:0.1 and 1:1). Aβ 1–42 fibril reduction at the pre-determined clinical dose of percepta (i.e. 1:0.1 wt/wt)^5^ caused a significant (p < 0.001) 45.3% disaggregation/reduction of pre-formed fibrils (Fig. 3B). Neuriva Original and neuriva Plus has some effects most likely due to the coffee cherry polyphenols in the dietary supplement but were not as effective on disaggregating/dissolving Aβ 1–42 fibrils on a wt/wt basis in comparison to percepta. Prevagen again demonstrated no effects on disaggregating/dissolving Aβ 1–42 fibrils on a wt/wt basis.

**Figure 4:**
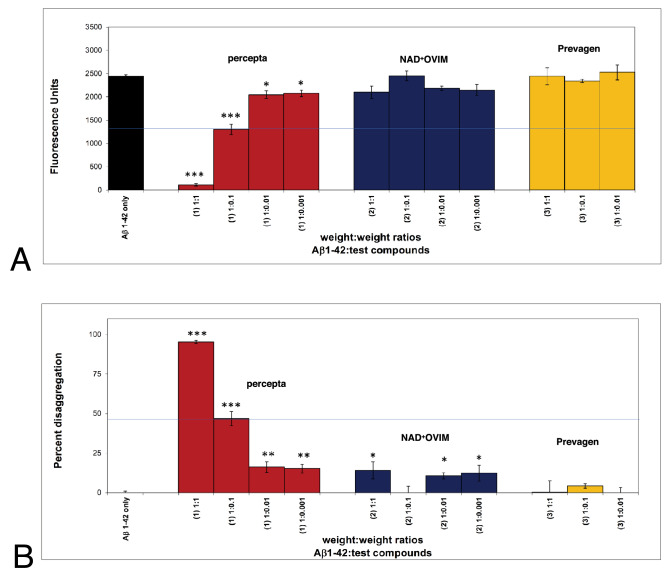
Percepta outperformed NAD^+^OVIM for disaggregation/disruption of pre-formed Aβ 1–42 fibrils. (**A**) Percepta was tested in comparison to NAD^+^OVIM and Prevagen supplements on a wt/wt basis. Percepta was the most potent disrupter of Aβ 1–42 fibrils in comparison to NAD^+^ OVIM and Prevagen on a wt/wt basis as quantitatively determined by Thioflavin T fluorometry. Aβ 1–42 fibrils demonstrate binding to Thioflavin T and 2,450 fluorescence units. Percepta demonstrated the most robust disaggregation/dissolution of pre-formed Aβ 1–42 fibrils. *p < 0.05, **p < 0.01, ***p < 0.001, by Student’s t-test. Bars represent mean ± SEM. (**B**) Comparison (on a wt/wt basis) of Percepta to NAD^+^ OVIM and Prevagen for % disaggregation of preformed Aβ 1–42 fibrils at four increasing dosages. Percepta had the most robust activity and reduced/disaggregated amyloid plaque fibrils in a dose-dependent manner (light brown bars). At a Aβ:percepta wt:wt ratio of 1:0.1 (blue line across graph), percepta disaggregated Aβ 1–42 fibrils by 48.4%, whereas NAD^+^ OVIM (that also contains cat’s claw) at that dose only disaggregated Aβ 1–42 fibrils by 13.3%, and Prevagen did not work on amyloid plaque fibrils at all. *p < 0.05, **p < 0.01, ***p < 0.001, by Student’s t-test. Bars represent mean ± SEM.

As shown in Fig. 4A and B, in a fourth comparison study, Aβ 1–42 first incubated at 37 °C demonstrated instant amyloid fibril formation by Thioflavin T fluorometry at day 0 (i.e. 2,450 fluorescent units; black bar on left). Percepta was again identified as the most potent disrupter of Aβ 1–42 fibrils in comparison to NAD^+^OVIM on a wt/wt basis and in a dose-dependent manner (Aβ 1–42:dietary supplement ratios tested of 1:0.001, 1:0.01; 1:0,1 and 1:1). Aβ 1–42 fibril reduction at the pre-determined clinical dose of percepta (i.e. 1:0.1 wt/wt)^5^ caused a significant (p < 0.001) 48.4% disaggregation/reduction of pre-formed fibrils (Fig. 4B). NAD^+^OVIM (even though it contains cat’s claw as an ingredient) was not effective at all in disaggregating/dissolving Aβ 1–42 fibrils on a wt/wt basis. Prevagen again demonstrated no effects on disaggregating/dissolving Aβ 1–42 fibrils on a wt/wt basis (Table 1).

The ranking order (criteria described in “Methods”) for memory-support supplement effects from the four studies on reducing Aβ 1–42 fibrils (Aβ 1–42: dietary supplement at 1:0.1 wt/wt) was percepta (69.6% reduction) >>> Alpha Brain (34.9%) = US Doctor’s Clinical Brain Power ADVANCED (32.4%) = BRAIN JUICE (30.1%) = neuriva Plus (27.0%) = neuriva Original (27.0%) > brainMD (NEUROVITE PLUS) (22.9%) = NOOCUBE (19.9%) = EXCELEROL (17.3%) = healthycell pro (17.2%) > Prevagen (12.9%) > PROCERA AVH (6.5%) = FOCUSfactor (5.5%) > Cebria (0%) = Brain Awake (0%) = Brain Support (0%) = brainMD (BRAIN & MEMORY POWER BOOST) (0%) = NAD^+^OVIM (0%) = BRAIN ARMOR (0%) = LUMONOL (0%) (Table 1).

**Figure 5:**
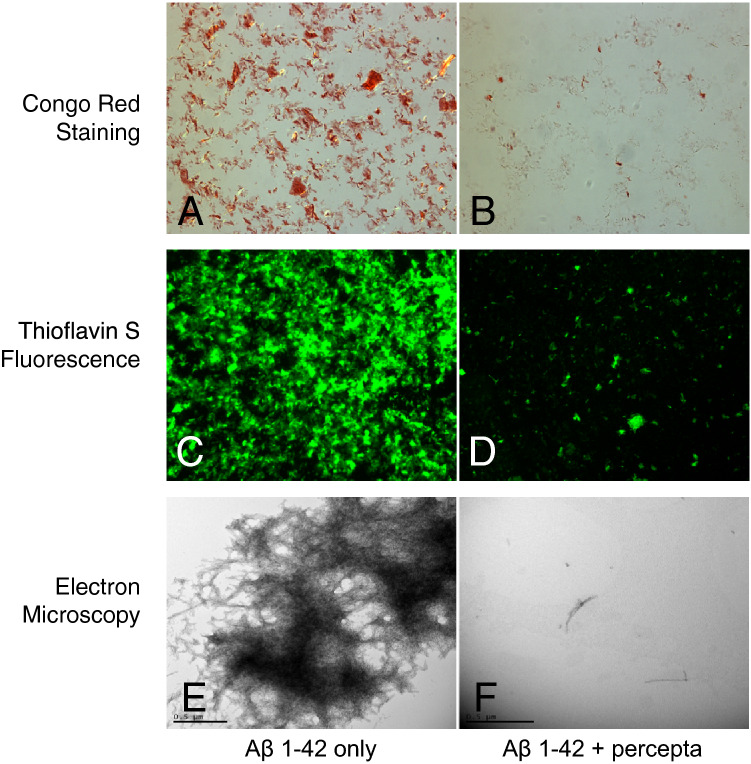
Percepta dissolved/disaggregated Aβ 1–42 fibrils within 24 h as shown by Congo red staining, Thioflavin S fluorescence and electron microscopy. (**A**) Aβ 1–42 fibrils show instant red-green birefringence indicative of Aβ 1–42 amyloid fibrils when stained with Congo red. (**B**) A marked dissolution/reduction of Congo red staining is observed in the presence of percepta. (**C**) Thioflavin S fluorescence showed massive Aβ 1–42 amyloid fibrils by positive green fluorescence. (**D**) A marked decrease in Thioflavin S fluorescence indicated that percepta dissolved Aβ 1–42 fibrils. (**E**) Negative stain electron microscopy demonstrated massive accumulation of Aβ 1–42 fibrils. (**F**). A marked dissolution/reduction of Aβ 1–42 fibrils was observed in the presence of percepta.

Figure 5 demonstrates the effects of percepta at a 1:1 wt/wt (Aβ 1–42:percepta) on reduction/dissolution of Aβ 1–42 fibrils by three different independent methods following 1 day of treatment. Aβ 1–42 fibrils show instant red-green birefringence indicative of amyloid fibrils when stained with Congo red (i.e. red/green birefringence as viewed under polarized light; Fig. 5A). A marked decrease in Congo red straining (i.e. red/green birefringence) was observed in the presence of percepta indicating a marked reduction of Aβ 1–42 fibrils (Fig. 5B). Thioflavin S fluorescence also demonstrated the presence of massive Aβ 1–42 fibrils as shown by positive green fluorescence (Fig. 5C). A marked decrease and near elimination in Thioflavin S fluorescence following incubation with percepta further demonstrated a marked reduction in Aβ 1–42 fibrils (Fig. 5D). Negative stain electron microscopy demonstrated massive accumulation of Aβ 1–42 fibrils as viewed at 30,000 magnification (Fig. 5E). A marked decrease /dissolution of Aβ 1–42 fibrils was observed at the electron microscopic level in the presence of percepta (Fig. 5F), confirming the reductions observed with Congo red staining and Thioflavin S fluorescence.

### Percepta was the most effective reducer of tau protein paired helical/straight filaments in comparison to 12 other major memory-support dietary supplements

**Figure 6:**
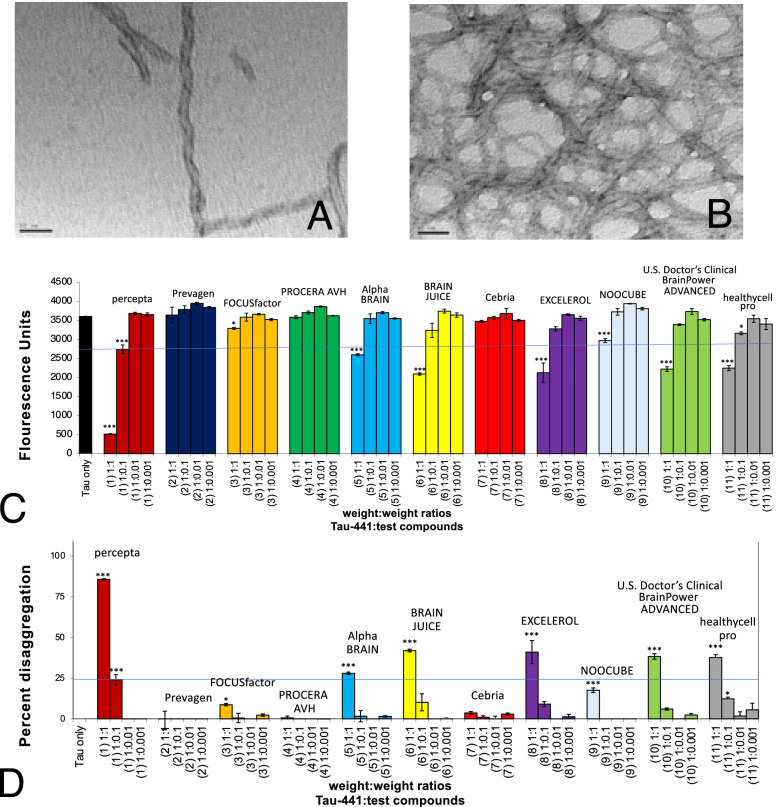
Percepta is the most potent disrupter of tau protein tangles in comparison to ten (10) other major brain supplements on a wt/wt basis. (**A**) Tau-441 induced by heparin forms paired helical filaments as shown by electron microscopy. Scale bar = 50 nm. (**B**) Formation of massive aggregated tau protein paired helical filaments following induction with heparin and shown by electron microscopy. Scale bar = 50 nm. (**C**) Percepta was the most potent disrupter /disaggregator of pre-formed tau protein tangles than any other memory-support supplement tested as shown quantitatively by Thioflavin T fluorometry. Tau protein-441 induced by heparin forms paired helical filaments instantly and shows 3600 Thioflavin T fluorescent units (FU)(black bar). Increasing doses of percepta (tau:percepta wt/wt ratios of 1:0.001, 1:0.01; 1:0.1; and 1:1) demonstrated a marked dose-dependent disaggregation/disruption of pre-formed tau tangles. *p < 0.05, **p < 0.01, ***p < 0.001, by Student’s t-test. Bars represent mean ± SEM. (**D**) Comparison of percepta to 10 (ten) other major memory-support dietary supplements in % disaggregation of preformed Aβ 1–42 fibrils. Percepta had the most robust activity and reduced/disaggregated tau tangles in a dose-dependent manner (light brown bars). Percepta disaggregated tau protein tangles by 86.4% at a tau:percepta wt/wt of 1:1, and by 24.1% at a tau:percepta wt/wt of 1:0.1, and much better than any other major memory-support dietary supplement tested including Prevagen, FOCUSFactor and Alpha Brain. *p < 0.05, **p < 0.01, ***p < 0.001, by Student’s t-test. Bars represent mean ± SEM.

**Figure 7:**
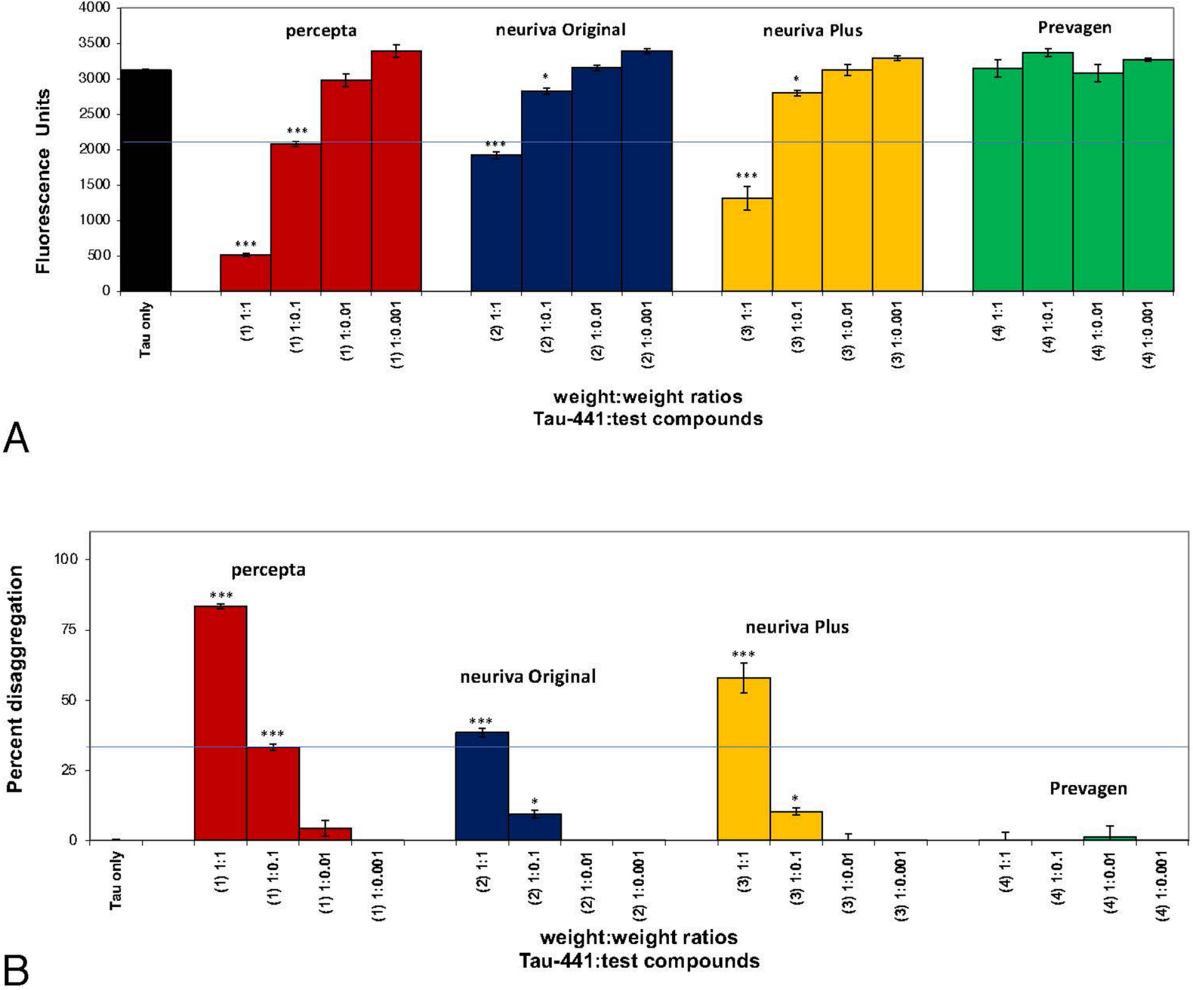
Percepta outperformed neuriva (Schiff Vitamins) for disaggregation /disruption of pre-formed tau tangles. (**A**) Percepta was tested in direct comparison to neuriva (neuriva Original and neuriva Plus) and Prevagen memory-support supplements on a wt/wt basis. Percepta was the most potent disrupter of tau tangles in comparison to neuriva (neuriva Original and neuriva Plus) and Prevagen on a wt/wt basis as determined quantitatively by Thioflavin T fluorometry. Tau protein tangles fibrils demonstrated binding to Thioflavin T and 3,120 fluorescence units. Percepta demonstrated the most robust disaggregation/dissolution of pre-formed tau tangles in a dose-dependent manner. *p < 0.05, **p < 0.01, ***p < 0.001, by Student’s t-test. Bars represent mean ± SEM. (**B**) Comparison (on a wt/wt basis) of percepta to neuriva (Original and Plus) and Prevagen for % disaggregation of pre-formed tau tangles at 4 increasing dosages. Percepta had the most robust activity and reduced/disaggregated tau tangles in a dose-dependent manner (light brown bars). At a tau:percepta wt:wt ratio of 1:0.1 (blue line across graph), percepta disaggregated tau tangles by 33.5%, whereas neuriva Original at that dose only disaggregated tau tangles by 8.1%, neuriva Plus only disaggregated tau tangles by 10.4%, and Prevagen did not work on reducing tau tangles at all. At a 1:1 wt/wt dose percepta reduced tau tangles by 85.7%, whereas neuriva Original and neuriva Plus only reduced tau tangles by 38.4% and 57.4%, respectively, whereas Prevagen had no effects at all. *p < 0.05, **p < 0.01, ***p < 0.001, by Student’s t-test. Bars represent mean ± SEM.

In two sets of tau tangle comparison studies, 13 different brain supplements were compared directly to each other on a wt/wt basis for their ability to disaggregate and reduce pre-formed tau paired helical and straight filaments (major constituents of neurofibrillary tangles). Testing was not determined for memory-support supplements that showed 0% efficacy for Aβ 1–42 fibrils including Brain Awake, Brain Support, brainMD, NAD^+^OVIM, BRAIN ARMOR, and LUMONOL. Memory-support supplements directly compared in a first set of studies were percepta, Prevagen, FOCUSfactor, PROCERA AVH, Alpha Brain (contains cat’s claw), BRAIN JUICE, Cebria, EXCELEROL, NOOCUBE (contains cat’s claw), US Doctor’s Clinical Brain Power ADVANCED, healthycell pro (contains cat’s claw) (Fig. 6C,D) and neuriva (neuriva Original and neuriva Plus) (Fig. 7A) (Table 1).

Figure 6A and B demonstrated the formation of paired helical and straight filaments after 3 days of incubation with Tau-441 with the highly sulfated glycosaminoglycan, heparin^5,32–37^. Paired helical filament formation appeared identical to that seen in brain aging and AD^5,32–37^. These pre-formed tangles help compare the effects of different memory-support supplements for disaggregation/reduction of pre-formed tau tangles using quantitative Thioflavin T fluorometry as described below.

As shown in Fig. 6C, tangle formation was confirmed by Thioflavin T fluorometry at day 3 to reach 3,600 fluorescent units (black bar on left). Percepta was identified as the most potent disrupter of tau protein tangles in comparison to the other ten (10) memory-support dietary supplements on a wt/wt basis and in a dose-dependent manner (tau: dietary supplement ratios tested of 1:0.001, 1:0.01; 1:0.1 and 1:1) (Table 1). Increasing doses of percepta demonstrated a significant dose-dependent disaggregation/disruption of pre-formed tau tangles at the two highest doses tested (i.e. 1:0.01 and 1:1 wt/wt) (Fig. 6C, D). Tau tangle reduction at 1:0.1 wt/wt clinical dose of percepta^5^ caused a significant (p < 0.001) 24.1% disaggregation/reduction of pre-formed fibrils (Fig. 6D), and a significant (p < 0.001) 85.7% disaggregation at 1:1 wt/wt (Fig. 6D). All of the other memory-support dietary supplements tested had little to no effects on disaggregation/reduction of pre-formed tangles, even those that contained cat’s claw as part of their ingredients (i.e. Alpha Brain, NOOCUBE and healthycell pro all contain cat’s claw) (Table 1).

As shown in Fig. 7A, in a separate study, tau protein induced by heparin demonstrated tangle formation as shown by a marked increase in Thioflavin T fluorometry to 3120 fluorescent units (black bar on left). Percepta was the most potent disrupter of tau tangles in comparison to neuriva Original, neuriva Plus and Prevagen on a wt/wt basis and in a dose-dependent manner (tau: dietary supplement wt/wt ratios tested of 1:0.001, 1:0.01; 1:0.1 and 1:1). Tau tangle disaggregation /dissolution at the pre-determined 1:0.1 wt/wt clinical dose of percepta^5^ caused a significant (p < 0.001) 33.5% disaggregation/reduction of pre-formed tau tangles, and a significant (p < 0.001) 83.4% disaggregation/dissolution at a 1:1 wt/wt (Fig. 7B) (Table 1). Neuriva Original and neuriva Plus has some effects but was more than 50% inferior to the reducing/disaggregation effects observed with percepta (Fig. 7B). Again, Prevagen had no effect at all in reducing pre-formed tau tangles (Fig. 7B).

The ranking order for memory support supplement effects on reducing tau paired helical/straight filaments (tau: dietary supplement at 1:1 wt/wt at 3 days) was percepta (85.7% reduction) >>> neuriva Plus (57.9%) >> BRAIN JUICE (41.9%) = EXCELEROL (41.0%) = neuriva Original (38.4%) = US Doctor’s Clinical Brain Power ADVANCED (38.3%) = healthycell pro (37.6%) >> Alpha Brain (27.9%) >> NOOCUBE (17.6%) >> FOCUSfactor (8.7%) > Cebria (3.6%) = PROCERA AVH (0%) = Prevagen (0%) (Table 1).

### Percepta demonstrated a potent reduction/dissolution of Aβ 1–42 fibrils and tau tangles as shown by electron microscopy and circular dichroism spectroscopy

**Figure 8:**
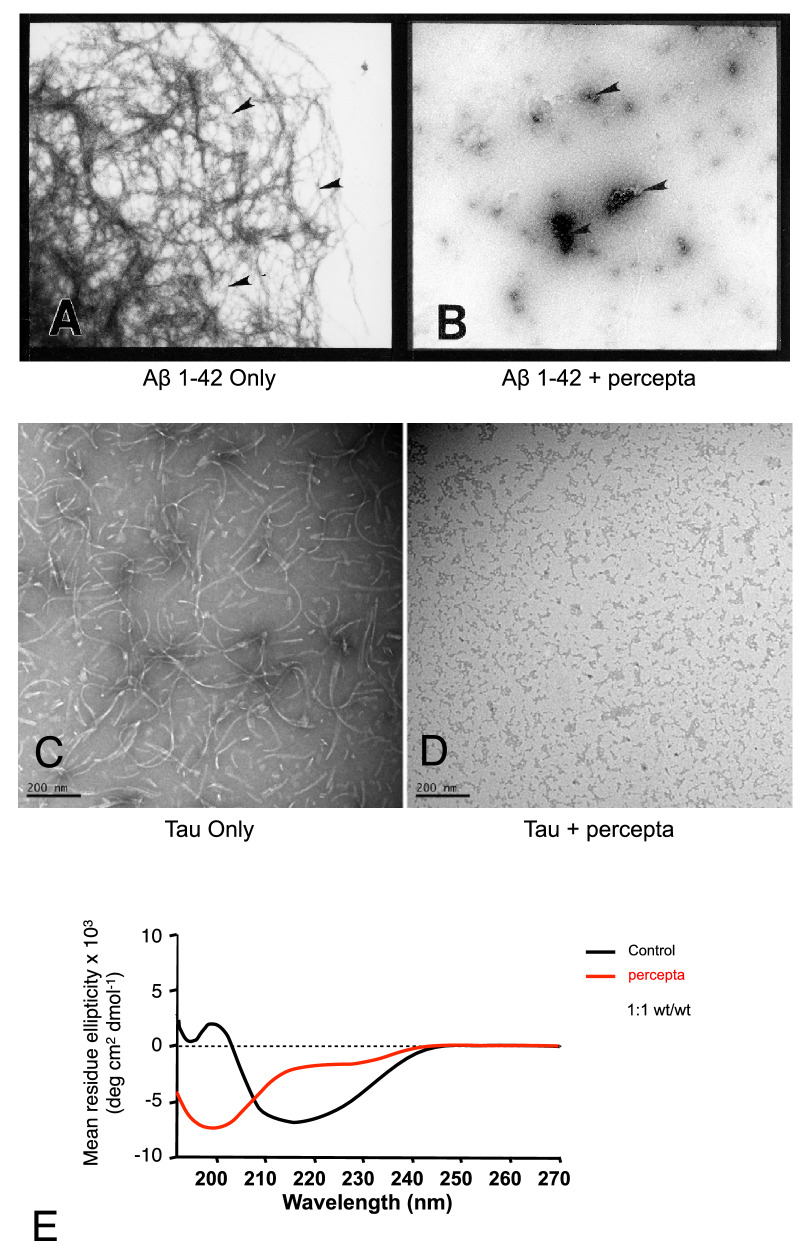
Disruption of Aβ 1–42 fibrils and tau protein tangles by percepta as shown by negative stain electron microscopy. (**A**) Aβ 1–42 fibrils (arrowheads) at 3 days as visualized by electron microscopy. × 30,000 magnification. (**B**) Aβ 1–42 fibrils in the presence of percepta following 3 days of co-incubation demonstrates only amorphous non-fibrillar material remaining (arrowheads). (**C**) Tau protein tangles formed by tau-441 induced in the presence of heparin for 3 days. Bar = 200 nm. (**D**) Marked dissolution/disaggregation of pre-formed tau protein tangles in the presence of percepta. Bar = 200 nm. (**E**) Percepta prevents formation of β-sheet tau protein tangles (shifts to α-helix) within 72 h as determined by CD spectroscopy.

Figure 8 demonstrates the effectiveness of percepta on reduction of both Aβ 1–42 fibrils and tau protein tangles at a 1:1 wt/wt ratio following co-incubation for 3 days as shown by electron microscopy. In Fig. 8A, Aβ 1–42 amyloid plaque fibrils (arrowheads) were visualized at high magnification (× 30,000). Aβ 1–42 amyloid fibrils in the presence of percepta following 3 days of co-incubation demonstrated only amorphous non-fibrillar material remaining (arrowheads) (Fig. 8B). Tau protein tangles formed by tau-441 induced in the presence of heparin for 3 days can also be visualized by electron microscopy (Fig. 8C). In the presence of percepta, a marked dissolution/disaggregation of pre-formed tau protein tangles was observed (Fig. 8D). A time course study indicated that percepta dissolved/disaggregated pre-formed tau tangles within 15 min of co-incubation (data not shown). Circular dichroism spectroscopy revealed that percepta prevented the formation of β-sheet tau protein tangles (shifted to α-helix) following co-incubation for 3 days (Fig. 8E). These studies confirm the reductive effects of percepta on both Aβ 1–42 fibrils and tau protein tangles.

### PTI-00703 Cat’s claw was the most effective reducer of Aβ 1–42 fibrils compared to 17 other manufacturers of Cat’s claw

**Figure 9:**
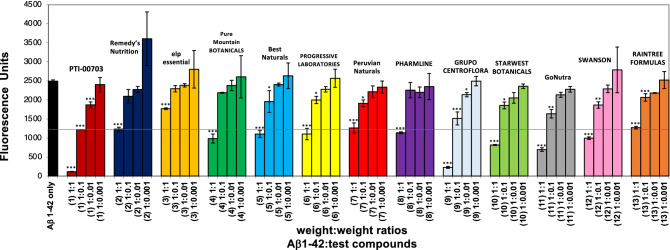
Identification of PTI-00703 Cat’s claw (*Uncaria tomentosa*) as the most potent disrupter of Aβ 1–42 fibrils in comparison to other cat’s claw sources in the marketplace today. PTI-00703 Cat’s claw compared to 12 other cat’s claw sources for its ability to disrupt/dissolve pre-formed Aβ 1–42 fibrils. This included cat’s claw from Remedy’s Nutrition, elp essential, Pure Mountain BOTANICALS, Best Naturals, PROGRESSIVE LABORATORIES, Peruvian Naturals, PHARMLINE, GRUPO CENTROFLORA, STARWEST BOTANICALS, GoNutra, SWANSON, and RAINTREE FORMULAS. PTI-00703 Cat’s claw demonstrated a dose-dependent disruption/disaggregation of pre-formed Aβ 1–42 fibrils. At the Aβ 1–42:PTI-00703 cat’s claw dose of 1:0.1 wt/wt there was a 51.4% dissolution of plaque fibrils (blue line across graph). No other cat’s claw extract demonstrated a similar effect at the 1:0.1 and 1:1 wt/wt doses like PTI-00703 Cat’s claw. *p < 0.05, **p < 0.01, ***p < 0.001, by Student’s t-test. Bars represent mean ± SEM.

PTI-00703 cat’s claw (from Cognitive Clarity Inc. and main ingredient in percepta) was also compared to 17 other cat’s claw sources for their ability to disrupt/dissolve pre-formed Aβ 1–42 amyloid plaque fibrils. In a first study, this included cat’s claw from Remedy’s Nutrition, elp essential, Pure Mountain BOTANICALS, Best Naturals, PROGRESSIVE LABORATORIES, Peruvian Naturals, PHARMLINE, GRUPO CENTROFLORA, STARWEST BOTANICALS, GoNutra, SWANSON, and RAINTREE FORMULAS. PTI-00703 cat’s claw (exclusive to percepta) demonstrated the most robust dose-dependent disruption/disaggregation of pre-formed Aβ 1–42 amyloid plaque fibrils in comparison to the other cat’s claw extracts tested on a wt/wt/basis (Fig. 9). At the clinical 1:0.1 wt/wt determined dose of Aβ 1–42:PTI-00703 cat’s claw^5^, there was a 51.4% reduction of Aβ 1–42 fibrils by PTI-00703 (Fig. 9; see blue line across graph). No other manufacturer of cat’s claw demonstrated a similar effect at the 1:01 and 1:1 wt/wt doses like PTI-00703 cat’s claw for disruption of pre-formed Aβ 1–42 fibrils (Fig. 9) (Table 2).

**Table 2:** Comparison of 18 cat’s claw extracts from different manufacturer’s for reduction of Aβ 1–42 fibril and/or tau tangles.

Cat’s claw extract/product name/Company	Major ingredients	% reduction of _A13_ 1–42 Fibrils (1:0.1 wt/wt)	% reduction of Tau Tangles (1:1 wt/wt)
PTI-00703 cat’s claw (Cognitive Clarity Inc.)	Cat’s claw (*Uncaria tomentosa*); 25 kg bags	51.2	90.1
GRUPO CENTROFLORA (Centroflora Group)	Cat’s claw (*Uncaria tomentosa*) bark	39.3	86.6
GoNutra (GoNutra)	Cat’s claw (*Uncaria tomentosa*) bark; bark powder in 1 lb bags	34.4	61.2
NOW (Now Foods)	Cat’s claw (*Uncaria tomentosa*) bark; 500 mg capsules	27.7	80.4
STARWEST BOTANICALS (Starwest Botanicals Inc.)	Cat’s claw (*Uncaria tomentosa*) bark; bark powder in 1	25.6	64.9
SWANSON (Swanson Health Products)	Cat’s claw (*Uncaria tomentosa*) bark; 500 mg capsules	25.1	45.2
Peruvian Naturals (Peruvian Naturals)	Cat’s claw (*Uncaria tomentosa*) bark; 500 mg capsules	23.4	25.1
SOLARAY (Solaray)	Cat’s claw (*Uncaria tomentosa*) bark; 500 mg capsules	23.3	62.0
Best Naturals (Best Naturals)	Cat’s claw (*Uncaria tomentosa*) bark; 500 mg capsules	21.5	48.7
PROGRESSIVE LABORATORIES (Progressive Laboratories)	Cat’s claw (*Uncaria tomentosa*) bark; 500 mg capsules	19.9	33.5
RAINTREE FORMULAS (Raintree Formulas)	Cat’s claw (*Uncaria tomentosa*) bark; 500 mg capsules	17.2	51.9
Herbal Plus (General Nutrition Corp.)	Cat’s claw (*Uncaria tomentosa*) bark; 500 mg capsules	16.1	45.1
Remedy’s Nutrition (Remedy’s Nutrition)	Cat’s claw (*Uncaria tomentosa*) bark 1000 mg capsules	15.9	60.2
Nature’s Way (Nature’s Way Products Inc)	Cat’s claw (*Uncaria tomentosa*) bark; 335 mg capsules	15.4	64.2
NATURE’S HERBS (Nature’s Herbs)	Cat’s claw (*Uncaria tomentosa*) bark; 500 mg capsules	13.5	64.6
Pure Mountain BOTANICALS (Pure Mountain Botanicals)	Cat’s claw (*Uncaria tomentosa*) bark; 500 mg capsules	12.2	51.0
PHARMLINE (Pharmline Inc.)	Cat’s claw (*Uncaria tomentosa*) bark 500 mg capsules	9.5	60.3
elp essential (Essential Products)	Cat’s claw (*Uncaria tomentosa*) bark; 700 mg capsules	8.0	20.2

Major ingredient details are also compared for each product.

**Figure 10:**
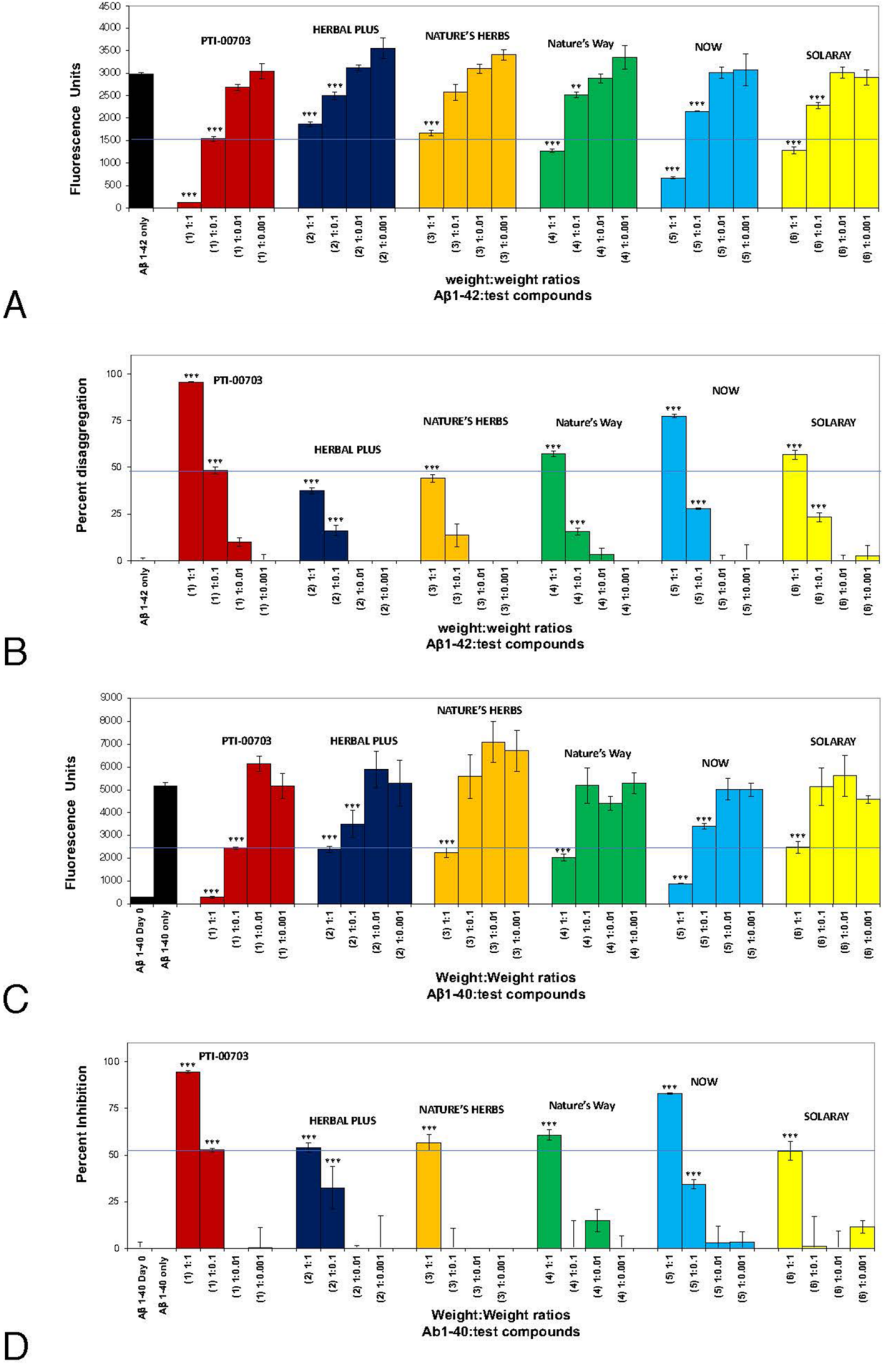
Comparison of PTI-00703 Cat’s claw to 5 other cat’s claw extracts. (**A**) Comparisons included cat’s claw from HERBAL PLUS, NATURE’S HERBS, Nature’s Way, NOW, and SOLARAY. PTI-00703 Cat’s claw demonstrated the most robust activity in disrupting/disaggregation of pre-formed Aβ 1–42 fibrils in a dose-dependent manner. At a 1:0.1 wt/wt PTI-00703 Cat’s claw demonstrated a 52.3% disaggregation/dissolution of pre-formed Aβ 1–42 fibrils, whereas the other cat’s claw extracts demonstrated much less activity (less than 20%) at that same dose. *p < 0.05, **p < 0.01, ***p < 0.001, by Student’s t-test. Bars represent mean ± SEM. (**B**) Disaggregation of preformed Aβ 1–42 fibrils by PTI-00703 Cat’s claw in comparison to other cat’s claw extracts. Comparisons were made between PTI-00703 Cat’s claw and cat’s claw extracts from Herbal Plus, Nature’s Herbs, Nature’s Way, NOW, and SOLARAY. At the 1:0.1 wt/wt dose of Aβ 1–42: PTI-00703 Cat’s claw significantly disaggregated Aβ 1–42 amyloid plaque fibrils by 48.6%. The next closest activity to that dose was cat’s claw from the cat’s claw product from NOW, in which there was just a 26.1% disaggregation. *p < 0.05, **p < 0.01, ***p < 0.001, by Student’s t-test. Bars represent mean ± SEM. (**C**) PTI-00703 Cat’s claw is a potent inhibitor of Aβ 1–40 fibril formation. Thioflavin T fluorometry compared PTI-00703 Cat’s claw to other cat’s claw extract (including HERBAL PLUS, NATURE’S HERBS, Nature’s Way, NOW, and SOLARAY) for inhibition of formation of Aβ 1–40 fibrils within a 3-day period. Aβ 1–40 peptide formed amyloid fibrils detected by Thioflavin T fluorometry within 3 days when incubated at 37 °C (black bars). (**D**) At the 1:0.1 wt/wt dose of Aβ 1–40: PTI-00703 Cat’s claw significantly inhibited Aβ 1–40 fibril formation by 52.3%. *p < 0.05, **p < 0.01, ***p < 0.001, by Student’s t-test. Bars represent mean ± SEM.

In a second study, PTI-00703 cat’s claw was compared to 5 other cat’s claw extracts for their ability to disaggregate/disrupt pre-formed Aβ 1–42 fibrils including those derived from HERBAL PLUS, NATURE’S HERBS, Nature’s Way, NOW, and SOLARAY. From these cat’s claw extracts tested, PTI-00703 cat’s claw again demonstrated the most robust activity in disrupting/disaggregation of pre-formed Aβ 1–42 fibrils in a dose-dependent manner, and in comparison to the other cat’s claw extracts tested (Fig. 10A,B). At a 1:0.1 wt/wt PTI-00703 cat’s claw demonstrated a 48.4% disaggregation/dissolution of pre-formed Aβ 1–42 fibrils, whereas the other cat’s claw extracts demonstrated much less activity (less than 20%) at that same dose (Fig. 10B).

The ranking order for cat’s claw (*Uncaria tomentosa*) effects on reducing Aβ 1–42 plaque fibrils (Aβ 1–42:cat’s claw extract at 1:0.1 wt/wt) was PTI-00703 cat’s claw (51.2% reduction) >> GRUPO CENTROFLORA (39.3%) = GoNutra (34.4%) > NOW (27.7%) = STARWEST BOTANICALS (25.6%) = SWANSON (25.1%) = Peruvian Naturals (23.4%) = SWANSON (23.3%) = Best Naturals (21.5%) = PROGRESSIVE LABORATORIES (19.9%) = RAINTREE FORMULAS (17.2%) = HERBAL PLUS (16.1%) = Remedy’s Nutrition (15.9%) = Nature’s Way (15.4%) = NATURE’S HERBS (13.5%) = Pure Mountain BOTANICALS (12.2%) > PHARMLINE (9.5%) = elp essential (8.0%) (Table 2). These studies also demonstrated that cat’s claw is a potent inhibitor in reducing Aβ 1–42 fibrils but there are marked differences in efficacy depending on the source of the cat’s claw with PTI-00703 being the most efficacious (Table 2).

### PTI-00703 Cat’s claw was the most potent inhibitor of beta-amyloid 1–40 fibril formation compared to 5 other manufacturers tested

In another study, Thioflavin T fluorometry compared PTI-00703 cat’s claw to other cat’s claw extracts (including HERBAL PLUS, NATURE’S HERBS, Nature’s Way, NOW, and SOLARAY) for their ability to inhibit formation of Aβ 1–40 fibrils within a 3-day period. Aβ 1–40 peptide formed amyloid fibrils detected by Thioflavin T fluorometry within 3 days (Fig. 10C,D). At the 1:0.1 wt/wt dose of Aβ 1–40: PTI-00703 cat’s claw significantly (p < 0.001) inhibited Aβ 1–40 fibril formation by 52.3%, and at the 1:1 wt/wt dose there was a significant (p < 0.001) 94.2% inhibition by PTI-00703 cat’s claw (Fig. 10D) . Cat’s claw extracts obtained from HERBAL PLUS, NATURE’S HERBS, Nature’s Way, NOW, and SOLARAY) were far inferior in inhibiting Aβ 1–40 fibril formation (Fig. 10D).

### PTI-00703 cat’s claw was the most potent reducer/disaggregator of tau protein tangles compared to cat’s claw extracts from 17 other manufacturers

**Figure 11:**
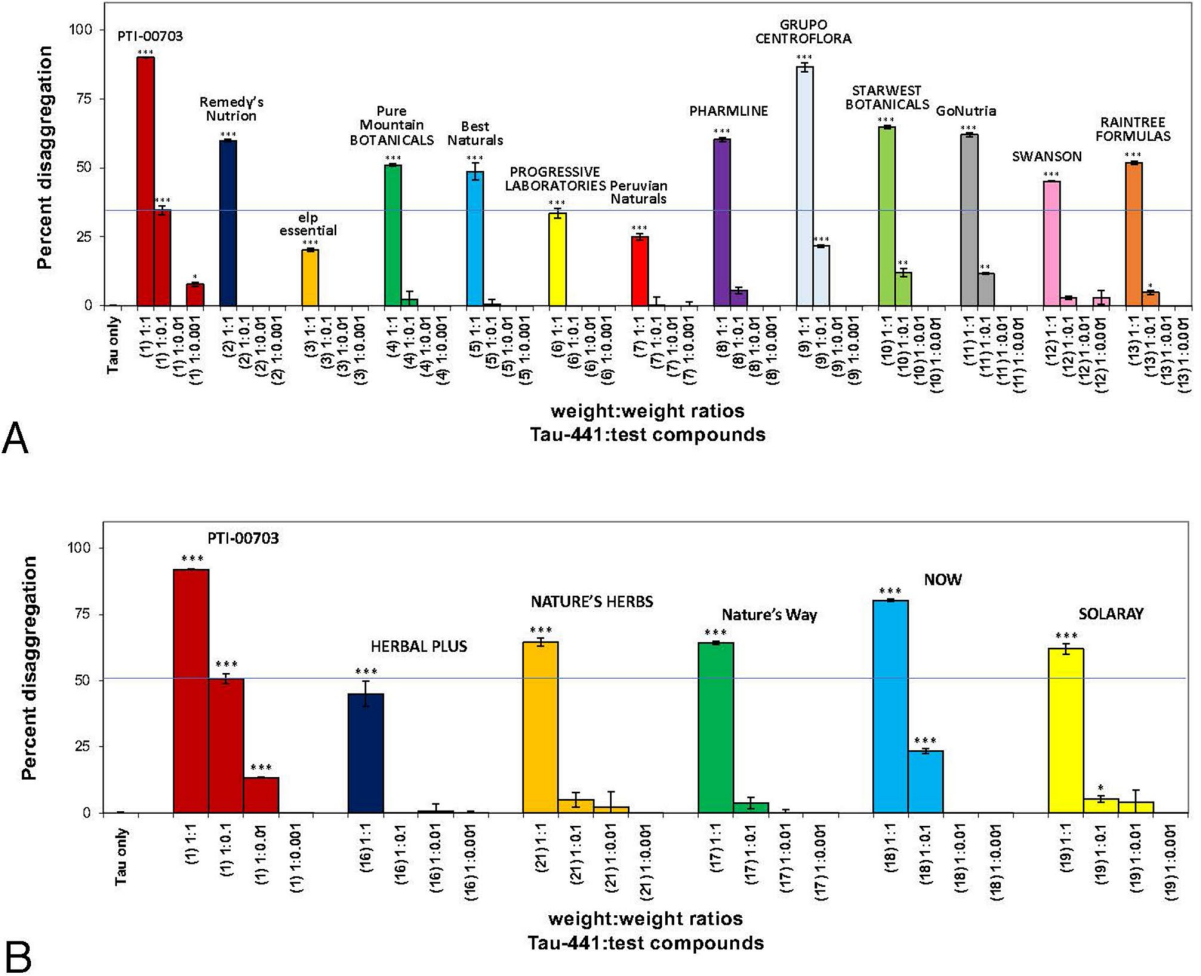
PTI-00703 Cat’s claw is the most potent disaggregator of tau tangles in comparison to 12 other cat’s claw extracts tested. (**A**) PTI-00703 Cat’s claw compared to other cat’s claw extracts for % disaggregation of pre-formed tau tangles induced by heparin following 3 days of treatment. Comparisons were made on a wt/wt basis between PTI-00703 Cat’s claw and cat’s claw extracts from Remedy’s Nutrition, elp essential, Pure Mountain BOTANICALS, Best Naturals, PROGRESSIVE LABORATORIES, Peruvian Naturals, PHARMLINE, GRUPO CENTROFLORA, STARWEST BOTANICALS, GoNutra, SWANSON, and RAINTREE FORMULAS. At the 1:0.1 wt/wt dose of tau protein: PTI-00703 Cat’s claw significantly disaggregated tau protein tangles by 34.3%. Cat’s claw extract from GRUPO CENTROFLORA was the closest to the robust activity demonstrated by PTI-00703 Cat’s claw with only 20.2% disaggregation shown at the 1:0.1 wt/wt dose. *p < 0.05, **p < 0.01, ***p < 0.001, by Student’s t-test. Bars represent mean ± SEM. (**B**) Comparisons of PTI-00703 Cat’s claw to 5 other cat’s claw extracts (including cat’s claw extracts obtained from HERBAL PLUS, NATURE’S HERBS, Nature’s Way, NOW, and SOLARAY). PTI-00703 Cat’s claw caused a robust dose-dependent disaggregation of pre-formed tau tangles, and much better than the other 5 cat’s claw extracts tested. At the 1:0.1 wt/wt dose of Tau-441: PTI-00703 Cat’s claw there was a 50.6% significantly disaggregation of tau protein tangles. *p < 0.05, **p < 0.01, ***p < 0.001, by Student’s t-test. Bars represent mean ± SEM.

Tau-441 protein was incubated with heparin for 3 days to induce tau tangle paired helical filament formation^5,35–37^. Thirteen (13) different cat’s claw extracts were then incubated for 3 days and compared on a wt/wt basis to determine their direct effects of reducing pre-formed tau tangles. As shown in Fig. 11A, PTI-00703 cat’s claw was the most potent disaggregator of tau tangles in comparison to 12 other cat’s claw extracts tested, and did so in a dose-dependent manner (Table 2). In this study, direct comparisons were made between PTI-00703 cat’s claw and cat’s claw extracts from Remedy’s Nutrition, elp essential, Pure Mountain BOTANICALS, Best Naturals, PROGRESSIVE LABORATORIES, Peruvian Naturals, PHARMLINE, GRUPO CENTROFLORA, STARWEST BOTANICALS, GoNutra, SWANSON, and RAINTREE FORMULAS. At the 1:0.1 wt/wt dose of tau protein: PTI-00703 cat’s claw significantly disaggregated tau protein tangles by 34.3%. (Fig. 11A) Cat’s claw extract from GRUPO CENTROFLORA was the closest to the robust activity demonstrated by PTI-00703 cat’s claw with only 21.3% disaggregation shown at the 1:0.1 wt/wt dose. (Fig. 11A). Comparisons of PTI-00703 cat’s claw in a separate study was also compared to 5 other cat’s claw extracts including those obtained from HERBAL PLUS, NATURE’S HERBS, Nature’s Way, NOW, and SOLARAY (Fig. 11B). Once again, PTI-00703 cat’s claw caused the most robust dose-dependent disaggregation of pre-formed tau protein tangles than each of the other 5 cat’s claw extracts tested (Fig. 11B). At the 1:0.1 wt/wt dose of Tau-441: PTI-00703 cat’s claw there was a significant (p < 0.001) 50.4% disaggregation of tau protein tangles. At the 1:1 wt/wt dose PTI-00703 cat’s claw significantly (p < 0.001) disaggregation of tau protein tangles by 92.3% (Table 2).

The ranking order for cat’s claw (*Uncaria tomentosa*) effects on reducing tau tangles (tau:cat’s claw extract at 1:1 wt/wt) was PTI-00703 cat’s claw (90.1% reduction) = GRUPO CENTROFLORA (86.6% ) > NOW (80.4%) >> STARWEST BOTANICALS (64.9%) = NATURE’S HERBS (64.6%) = Nature’s Way (64.2%) = SOLARAY (62.0%) = GoNutra (61.2%) = PHARMLINE (60.3%) = Remedy’s Nutrition (60.2%) > RAINTREE FORMULAS (51.9%) = Pure Mountain BOTANICALS (51.0%) = Best Naturals (48.7%) = SWANSON (45.2%) >> PROGRESSIVE LABORATORIES (33.5%) > Peruvian Naturals (25.1%) = elp essential (20.2%) (Table 2). These studies demonstrated that cat’s claw is a potent inhibitor in reducing tau tangles but there are marked differences in efficacy depending on the source (i.e. different manufacturers) of cat’s claw (Table 2).

At the tau:cat’s claw extract at 1:0.1 wt/wt, PTI-00703 cat’s claw reduced tau tangles by 30.2%, whereas GRUPO CENTROFLORA reduced tangles by 15.3% (Fig. 11A) indicating that PTI-00703 cat’s claw is double the efficacy of GRUPO CENTROFLORA cat’s claw at the more clinical dose of 1:0.1 wt/wt^5^.

## Discussion

### Causes of memory loss

Studies over the last 20 years now have determined that memory loss is primarily caused by the accumulation in brain of three major factors, **P**laques, **T**angles and **I**nflammation (i.e. **PTI**)^1,2^. Plaques look like giant Swedish-meatballs in the brain between neurons (i.e. nerve cells) and consist primarily of a 42-amino acid peptide known as beta-amyloid protein or Aβ^38,39^. Brain tangles look liked dried-up spaghetti inside neurons consisting of tau protein, a microtubule associated protein that normally functions in modulating the stability of axonal microtubules^3^. Both brain plaques and tangles are neurotoxic and cause neurons to die^40^. Neuroinflammation is the third factor that accelerates and worsens the clinical manifestations of memory loss^13,41,42^.

Aβ amyloid plaques in brain start accumulating in one’s early 20’s^6,43,44^. In Down’s syndrome (trisomy 21), Aβ amyloid accumulation occurs inside neurons as soon as 1 day after birth^26^, and Down’s syndrome patients end up having much of the same pathology as observed in human Alzheimer’s disease (AD) brain^26^. Tau protein tangles accumulate in brain shortly after Aβ plaque accumulation^45,46^. In seminal papers, organoids consisting of human stem cells from patients with familial AD were placed on a Matrigel matrix (consisting of extracellular matrix components) to shortly observe within weeks the formation of Aβ amyloid plaques. Tau protein tangles appeared a few weeks later demonstrating that plaques accumulate first in brain, followed by tangles^45,46^. These studies have been confirmed in other models^4^**.** Studies also showed that neuroinflammation causes massive neuronal death and severe memory loss usually leading to the diagnosis of AD^4,13^.

There have been instances of individuals whose brains are filled with plaques and tangles, and yet they did not show any clinical signs of memory loss. It turns out that the missing piece for them was the absence of neuroinflammation^47^. People who have brain plaques, tangles and inflammation all show the clinical signs of memory loss and all will develop AD, if they have not already done so^8,9,48^.

It is clear that there are many promising drugs in human clinical trials to target brain plaques, tangles and/or neuroinflammation^4^ to help support or even ameliorate the signs of cognitive decline and memory loss. Brain supplements to improve or sustain overall brain health and memory support is a fast growing industry, and there is an urgent need to help people today as they wait for an eventual drug or drugs to help alleviate memory loss for the long run. Brain supplements that help support memory are important products to fill the current need. All natural and plant-based products also appear to have less side effects than drugs, as they seem to already work in nature but need to be examined more closely.

One would think that dietary supplements for memory support and enhancement that are produced and placed in the marketplace would be targeting the major causes of memory loss—including brain plaques, tangles and neuroinflammation^20^. But as the present investigation demonstrates, most of them do not.

### Comparison of memory-support dietary supplements for effects on Aβ fibrils and tau tangles: polyphenols and cat’s claw appear to be key

The purpose of the present investigation was to implement a direct comparison of major memory-support dietary supplements in the marketplace today and to determine their potential effects on reducing brain Aβ 1–42 fibrils (i.e. the major component of brain plaques) and tau protein tangles—two of the three most important targets for memory loss. In our studies, quantitative Thioflavin T fluorometry (the gold standard for quantitation of amyloid/tau fibril and formation)^21–23^ were implemented to decipher which memory-support dietary supplements actually target and reduce/inhibit brain plaques (i.e. Aβ 1–42 fibrils) and tangles (i.e. paired helical and straight filaments). In addition, qualitative Congo red staining (i.e. red/green birefringence as viewed under polarized light)^24^, Thioflavin S fluorescence^25^, negative stain electron microscopy and/or CD spectroscopy were used to confirm the results obtained with the most effective supplement identified, percepta. The studies were implemented on memory-support dietary supplements purchased from a variety of sources including amazon.com, other internet websites, and mainstream retail stores.

Of all the products tested, percepta (consisting of PTI-00703 cat’s claw and a specific oolong tea extract) was the most effective brain dietary supplement in specifically targeting and reducing/disaggregating and inhibiting brain Aβ fibrils and tau tangles. This including its remarkable effects on reducing/disaggregating brain Aβ 1–42 fibrils (21 supplements tested) and tau tangles (13 supplements tested). Percepta turned out to be minimally 25–89% significantly more effective in reducing pre-formed Aβ fibrils and tau tangles in comparison to other tested major memory-support dietary supplements.

The results were not skewed by solubility issues as each of the dietary supplements were successfully diluted into increasing concentrations for quantitative Thioflavin T fluorometry comparisons. In addition, no quenching (background readings of compounds only) was observed using colored compounds, and any fluorescence given off by any of the compounds in the presence of the Thioflavin T reagent was always subtracted from all pertinent readings^21–23^_._

We hypothesize that many of the positive Aβ 1–42 fibril and tau tangles reducing effects of other memory-support supplements were due to the presence of specific polyphenols as part of the final formulations. For example, US Doctor’s Clinical Brain Power ADVANCED reduced Aβ 1–42 fibrils by 32.0% (1:0.1 wt/wt) and tau tangles by 38.3% (1:1 wt/wt) likely due to the presence of green tea in their formulation (Table 1). Other memory-support products demonstrating reduction of Aβ 1–42 fibrils and/or tau tangles with polyphenols in their formulation include BRAIN JUICE (green tea, blueberries), neuriva Plus and neuriva Original (coffee fruit polyphenols), and EXCELEROL (green tea, white tea, black tea). Previous studies have demonstrated that tea polyphenols are good inhibitors/reducers of Aβ 1–42 fibrils and/or tau tangles^49–51^. Most interesting many of the high positive Aβ 1–42 fibril and tau tangles reducing effects by memory-support supplements was likely due to the presence of cat’s claw including percepta, Alpha Brain, NOOCUBE, and healthycell pro (Table 1). PTI-00703 cat’s claw was found by Snow et al.^5^ to contain many important polyphenols in its composition including epictechin, chlorogenic acid, and proanthocyanidins (i.e. epicatechin dimers and trimers). The proanthocyanidins (as found in grape seed extract; red wine, berries) are believed to be the most potent inhibitors and reducers of Aβ 1–42 fibril and tau tangles as previously determined^5^, and as confirmed in the present investigation.

Many of the memory-support supplements that we tested contain multiple ingredients from 10–20 + ingredients all formulated together into large or multiple capsules/pills that the consumer is required to take as a daily serving. We found that increasing the total amount of different ingredients into a single capsule leads to the blocking of efficacy of individual ingredients when combined into a large mixture (Snow et al., unpublished data). Thus, much of the efficacy observed with single ingredients will be diminished substantially by adding more ingredients into a daily serving dose, and many of the ingredients will end up being blocked from even entering the brain, and therefore would have little to no efficacy as a memory-support dietary supplement. Most manufacturers of dietary supplement combinations are unaware of these blocking effects that we have observed.

### Percepta is the most effective reducer of Aβ fibrils and tau tangles due to PTI-00703 cat’s claw

Percepta dissolved/reduced Aβ fibrils rather quickly (nearly instantly) as demonstrated by loss of Congo red staining (red/green birefringence as viewed under polarized light), Thioflavin S fluorescence, and fibrils as visualized by electron microscopy (Fig. 5). Tau tangles were also disaggregated/dissolved in the presence of percepta and did so within 15 min of interaction. Circular dichroism spectroscopy deciphered the mechanism in that percepta was effective in reducing and inhibiting β-sheet tau PHFs into α-helix or amorphous material. Previous studies also demonstrated that PTI-00703 cat’s claw (the main ingredient in percepta) reduced the beta-sheet secondary folding of both Aβ fibrils and tau tangles^5^. This disaggregation into more soluble amorphous material was also not believed to be neurotoxic as indicated in our previous studies^5^.

Many of the memory-support dietary supplements in the marketplace today contains the same ingredients such as vitamin B6, B12, phosphatidylserine (PD), ginkgo biloba, huperzine A, green tea extract, L-theanine and vinpocetine. Percepta, on the other hand, consists of just two major ingredients, PTI-00703 cat’s claw and a specific oolong tea extract. Cat’s claw is a woody vine that grows in the Amazon rain forest up to 200 feet in length^52,53^ and is referred as “cat’s claw” or “Una de Gato” due to its curved-like thorns^5^. Once it is harvested, the bark regrows to allow for a near endless supply of this important plant in the Amazon rain forests (Peru and Brazil). Its main genus is *Uncaria*. Although there are 34 species of cat’s claw, *Uncaria tomentosa* is the most widely used and sold in the Peruvian marketplaces, and initially was used for enhancement of the immune system as shown by Keplinger in the late 1980’s^54,55^.

In the early 2000’s, Snow et al.^5^ initially observed cat’s claw effects, first on Aβ brain plaque fibrils and then years later (when appropriate screening models were available) on tau tangle filaments. In plaque-producing TASD-41 transgenic mice, the main ingredient in PTI-00703 cat’s claw extract also markedly reduced neuroinflammation (both astrocytosis and microgliosis). In Feb 2019, Snow et al.^5^ published a 108-page paper in *Scientific Reports* in which new polyphenols (specific proanthocyanidins consisting of epicatechin-dimers) were identified responsible for the potent plaque and tangle reducing and inhibitory activity in PTI-00703 cat’s claw . The polyphenols of PTI-00703 cat’s claw entered the brain in 2-min (into brain parenchyma and cortex as shown by radiolabeling studies) and were believed responsible for the observed plaque, tangle and neuroinflammation reducing activity^5^.

The cat’s claw specifically used in these studies was named “**PTI**-00703 cat’s claw” since it seemed to work on the major causes of memory loss—**P**laques, **T**angles and **I**nflammation. It was obtained from a Peruvian source in which they harvested the bark from the plant 1–2 miles deep in the Peruvian Amazon rain forest. PTI-00703 cat’s claw also was derived from a unique proprietary isolation and concentration method that resulted in the most robust plaque and tangle reducing and inhibitory activity ever observed.

Previous studies demonstrated that one of the main ingredients in PTI-00703 cat’s claw (i.e. proanthocyanidin B2) markedly reduced brain plaques in TASD-41 double mutation plaque-producing mice (by 57%) and neuroinflammation (by 69–80%) in 3 months of treatment^5^. This was concurrent with a significant (p < 0.001) 58% improvement in short-term memory as assessed by Morris water maze testing^5^. Reduction of both Aβ 1–40 and Aβ 1–42 soluble and insoluble levels in brain was also observed in TASD-41 transgenic mouse brain following 3 months of treatment as assessed using quantitative ELISA’s from brain tissue^5^. This confirmed that the formation of off-pathway soluble oligomers are not toxic to brain cells and are believed to be cleared/eliminated by microglia in the brain^5^. The reduction in neuroinflammation by cat’s claw’s is also probably caused by its known ability to markedly reduce the inflammatory cytokines interleukin-1 and TNF-α^27–30^.

The present investigation also identified PTI-00703 cat’s claw as the most effective cat’s claw for reducing Aβ 1–42 fibrils and tau tangles in comparison to 17 other manufacturers of cat’s claw. This is likely due to the source of cat’s claw which can be obtained from many parts of the world including the Peruvian and Brazilian rain forests. The elevation of the cat’s claw plant (i.e. source from where it is obtained) and how it is extracted/purified appears to change the final polyphenol content of the cat’s claw which will directly affect its ability to inhibit and reduce brain plaques (i.e. Aβ fibrils) and tangles (tau tangles) as previously described^5^. The source and extraction process to obtain PTI-00703 cat’s claw appears to contribute to the potent Aβ fibril and tau tangle activity observed (Table 2).

The inclusion of a specific oolong tea extract in percepta (known as MemorTea) is believed to contribute to the overall potent Aβ fibril and tau tangle inhibitory and reducing activity. Oolong tea, like green tea and black tea all contain potent ingredients like epicatechin, catechin, ECG and ECGC that are also believed to contribute to plaque and tangle reduction to some extent as previous studies have also shown^49–51,56,57^. However, we have discovered that partially fermented (i.e. partially oxidized) oolong tea has a more potent plaque and tangle inhibitory/reducing profile than green tea (i.e. unfermented or unoxidized) or black tea (fully fermented or fully oxidized) even through all are derived from the same plant (*Camellia sinensis*)^58,59^.

To conclude, our investigations demonstrate that of major memory-support brain dietary supplements in the marketplace today, the most effective to target and reduce Aβ 1–42 fibrils and tau tangles is percepta (a dietary supplement just containing two major ingredients, PTI-00703 cat’s claw and a specific oolong tea extract). Further in vivo animal studies and human clinical trials are anticipated to confirm these exciting and important observations.

## Methods

### 21 memory support supplements tested

Up to 21 top-selling dietary supplements were tested for their effects on reduction and/or inhibition of Aβ 1–42 fibrils (21 memory-support dietary supplements tested) and tau tangle paired helical/straight filaments (13 memory-support dietary supplements tested). These included the following brain memory support supplements: (1) Prevagen (Batch #1511036M; purchased online from www.amazon.com), (2) FOCUSFactor (Lot **#** 855119 006203 purchased online from amazon.com), (3) PROCERA AVH; Batch #5352IP668; purchased online from amazon.com), (4) Alpha Brain (Serial #1944401018; purchased online from www.onnit.com/alpha-brain), (5) NAD^+^OVIM Lot #192943S; purchased online from www.nadovim.com, (6) BRAIN JUICE (Batch #029692N001-W; samples obtained from Natural Products West tradeshow booth in Anaheim, CA, 2018); (7) Cebria (Lot #M159230; purchased at GNC store in Seattle, WA); (8) EXCELEROL (Lot #6535; purchased online from amazon.com), (9) NOOCUBE (Batch #17098; purchased online from amazon.com), (10) U.S. Doctors Clinical Brain Power ADVANCED (Lot # T14LO98; purchased at Rite Aid store, Lynnwood, WA), (11) healthycell pro (Batch #0226L6; purchased online from healthycell.com/healthycell-pro), (12) LUMONOL (Lot # 804043; purchased online from amazon.com), (13) Brain Awake (Lot # S18A008; purchased online from amazon.com), (14) BRAIN ARMOR (Lot #C1800717; purchased online from amazon.com), (15) brainMD—BRAIN & MEMORY POWER BOOST (Lot # 1324949; purchased online at brainmd.com/supplements), (16) Brain Support (Lot # 17606000; purchased online at amazon.com), 17) Clarity (Lot # HBE80421-30; purchased online at amazon.com), (18) brainMD: NEUROVITE PLUS (Lot # 1314676; purchased online at brainmd.com/supplements), (19) neuriva Original (Lot # US0509A; purchased at Rite Aid store, Lynnwood, WA); (20) neuriva Plus (Lot # US0499B; purchased at Rite Aid store, Lynnwood, WA, (21) percepta (Lot # 0716J7; purchased online at www.perceptabrain.com). Table 1 shows each memory-support dietary supplement, its source, major active ingredients, reducing effects on Aβ 1–42 fibrils and tau tangles, solubility issues and reported mechanisms of action/claims for each product.

### 18 Cat’s claw extracts tested

Up to 18 different cat’s claw extracts were also tested for their effects on reduction and/or inhibition of Aβ amyloid plaque fibrils and tau tangle paired helical/straight filaments. These included the following cat’s claw extracts: (1) Remedy’s Nutrition (cat’s claw bark -*Uncaria tomentosa*; Serial #98295 04839; purchased online at amazon.com), (2) elp essential (cat’s claw bark, *Uncaria tomentosa*; Lot #24028; 700 mg capsules; purchased online at amazon.com), (3) Pure Mountain BOTANICALS (cat’s claw bark, *Uncaria tomentosa*; Lot #201845115; 500 mg capsules; purchased online at amazon.com), 4) Best Naturals (cat’s claw bark, *Uncaria tomentosa*; Lot #101642; 500 mg capsules; purchased online at amazon.com), 5) PROGRESSIVE LABORATORIES (cat’s claw bark, *Uncaria tomentosa*; Lot #2116356; 500 mg capsules; purchased online at amazon.com), 6) Peruvian Naturals (cat’s claw bark, *Uncaria tomentosa*; Lot # 1101466; 500 mg capsules; purchased online at amazon.com); 7) PHARMLINE (cat’s claw, bark *Uncaria tomentosa*; Lot #M-3137; purchased online at amazon.com), 8) GRUPO CENTROFLORA (cat’s claw bark, *Uncaria tomentosa*; Batch #190607.2173; purchased online at Centroflora.com); 9) STARWEST BOTANICAL’S (cat’s claw bark, *Uncaria tomentosa*; bark powder in 1 lb bag; Item #201208-51; purchased online at amazon.com; 10) GoNutra (cat’s claw bark, *Uncaria tomentosa*; bark powder in 1 lb bag; Lot # PE0052518-005; purchased online at amazon.com), 11) SWANSON (cat’s claw root, *Uncaria tomentosa*; Batch #B 231101; purchased online at amazon.com), 12) RAINTREE FORMULAS (cat’s claw bark, *Uncaria tomentosa*; Lot #711029; purchased online at amazon.com); 13) HERBAL PLUS (cat’s claw bark, *Uncaria tomentosa*; Lot #5123HM1944; 500 mg capsules; purchase at GNC, Alderwood Mall, Lynnwood, WA), 14) NATURE’S HERBS (cat’s claw bark, *Uncaria tomentosa*, Lot #210412; 500 mg capsules; purchased online at amazon.com), 15) Nature’s Way (cat’s claw bark, *Uncaria* tomentosa; Batch # 20090763; 335 mg capsules), 16) NOW (cat’s claw bark, *Uncaria tomentosa*; Lot # 1451190 0956; 500 mg capsules; purchased online at iherb.com), 17) SOLARAY (cat’s claw bark, *Uncaria tomentosa*; Lot #160611; 500 mg capsules; purchased online at swansonvitamins.com), 18) PTI-00703 cat’s claw (Cognitive Clarity Inc. through exclusive third-party supplier from Lima, Peru; cat’s claw bark, *Uncaria tomentosa*, Lot # 1090294, 500 kg extracts in 25 kg double ethylene bags imported from Peru). Table 2 shows each cat’s claw extract/supplement, its source, major active ingredients, reducing effects on Aβ 1–42 fibrils and tau tangles, and solubility issues for each product.

### Isolation of Cat’s claw

PTI-00703 cat’s claw represents a specific dried extract of cat’s claw (*Uncaria tomentosa*) extracted by a specific manufacturing source in Peru. The general extraction process was previously described^5^.

### Thioflavin T fluorometry studies

The effects of different memory-support dietary supplements/products and cat’s claw (bark powder, *Uncaria tomentosa*) extracts to disaggregate/reduce pre-formed Aβ 1–42 fibrils and tau protein tangles, was determined using a previously described method of quantitative Thioflavin T fluorometry^21–23^. Using this sensitive quantitative assay, any decreases or increases in fluorescence correlated with an increase or decrease in the amount of Aβ fibrils or tau protein tangles present, allowing one to determine the extent of potential inhibitors and reducers of Aβ amyloid fibrils and/or tau protein tangles^60,61^.

For the studies involving Aβ fibrils, 22 µM of Aβ 1–40 or Aβ 1–42 (rPeptide, Watkinsville, GA, USA) was incubated in 96-well assay plates at 37 °C for 1–3 days (in triplicate) either alone or in the presence of increasing amounts of memory-support dietary supplement products (powder derived from contents of capsules, ground up tablets, or dried liquid) and/or cat’s claw bark powder extracts (i.e. test compounds). The Aβ:test compound ratios studied were usually 3–4 increasing concentrations at 1:0.001, 1:0.01, 1:0.1 and 1:1 ratios on a weight-to-weight basis. No real solubility issues of dietary supplement products were observed due to the high dilutions used from stock solutions.

Aliquots were taken and analyzed usually at 0, 1, 3 and 7 days of incubation. Following the incubation period, 240 µl of Aβ 1–40 or Aβ 1–42 ± increasing concentrations of memory- support dietary supplements (i.e. test compounds) and/or cat’s claw bark powder extracts were added to 60 µl of 500 µM Thioflavin T (Sigma Chemical Co., St. Louis, MO) in 50 mM NaPO_4_. Studies indicated that increasing concentrations of Aβ gave a proportional increase in the fluorescence in the presence of 500 µM Thioflavin T, ruling out the presence of any disproportionate inner filter effects. Most results reported represented 3 days of co-incubation with Aβ fibrils and tau tangles, and similar effects were observed at 7 days. % disaggregation was proportional to % reduction of Aβ 1–42 fibrils, and represented a disaggregation of beta-sheet secondary folding as we have previously shown with PTI-00703 cat’s claw assessed using circular dichroism spectroscopy^5^.

For the studies involving tau tangle paired helical filament/straight filament formation and disaggregation/reduction studies, 1 mg/ml (30 µg) tau-441 (rPeptide, Watkinsville, GA, USA) was first incubated with 10 mg/ml of heparin (porcine intestinal mucosa; Sigma Aldrich, St. Louis, MO) shaking at 500 rpm at 37 °C for 3 days to induce PHF formation as previous described^5^. Tau protein PHF formation was confirmed by positive Congo red staining (i.e. red/green birefringence under polarized right), Thioflavin S fluorescence (positive green fluorescence), and electron microscopy (visualization of paired helical filaments at 30,000 × magnification) (see Fig. 6A,B). Following the 3-day incubation period to induce paired helical filament formation, 200 µl (20 µg) of tau-441 heparin-induced PHFs were incubated in 96-well assay plates at 37 °C for 1–3 days (in triplicate) either alone or in the presence of increasing amounts of memory- support dietary supplement products (powder derived from contents of capsules, ground up tablets, or dried liquid) and/or cat’s claw bark powder extracts (i.e. test compounds). The tau:test compound ratios studied were usually at 1:0.001, 1:0.01, 1:0.1 and 1:1 ratios on a weight-to-weight basis. Data at 3 days was reported, with 7-day time points demonstrating similar effects.

Fluorescence emission at 485 nm was measured on a Molecular Devices instrument model SpectraMax GeminiXS fluorometer at an excitation wavelength of 450 nm. All fluorescence determinations were based on these references and any fluorescence given off by any of the dietary supplement products in the presence of the Thioflavin T reagent was always subtracted from all the pertinent readings. No quenching by colored compounds was observed at the concentrations/dilutions used. There was an excellent correlation between the findings found with Thiolfavin T fluorometry (i.e. like reduction of Aβ fibrils with Congo red staining, Thioflavin S fluorescence, and negative stain electron microscopy; see below).

### Blinded studies

All studies were implemented using blind-coding for each memory-support supplement product and cat’s claw extract/product. The research scientists implementing the studies were blinded to each code. The data was only unblinded by a third-party once all the data was gathered following the completion of all studies.

### Congo red staining

The presence of Aβ fibrils or tau tangles (which both consist of predominantly beta-sheet secondary structure) was assessed by Congo red staining (i.e. red/green birefringence as viewed under polarized light^24^. Briefly, 15 µl aliquots of incubated solutions [(Aβ 1–42 or tau-induced paired helical filaments in the absence or presence of extracts from memory-support dietary supplements or cat’s claw bark powder (i.e. *Uncaria tomentosa*) extracts] were reacted in solution with 5 µl of a 0.5% Congo red aqueous solution for 10 min. The samples were then micro-centrifuged for 2 min at high speed. Next 10 µl of the Congo red supernatant was pipetted off the sample tubes and 10 µl of double-distilled water was added. The samples were gently mixed and microcentrifuged for 2 min, and 15 µl of the Congo red supernatant was pipetted from each tube. The remaining material was well mixed and applied to slide wells (PTFE printed slides, EMS Sciences, Hatfield, PA). 3 µl of Vector Mount (Vector Labs, Burlingame, CA) was then added to the wells and cover slipped with 5 mm Phototech glass coverslips. The samples were viewed under polarized light and a prominent red/green birefringence was indicative of Aβ amyloid fibrils and tau protein paired helical and/or straight filaments. A reduction or loss of red/green birefringence staining was indicative of disaggregation/reduction of Aβ amyloid fibrils or tau tangles^5^.

### Thioflavin S fluorescence

The presence of Aβ amyloid fibrils and/or tau tangles was also assessed by Thioflavin S fluorescence as viewed under fluorescent light^25^. Briefly, 10–20 µl aliquots of incubated solutions (i.e. Aβ 1–42, tau-441 induced by heparin ± test memory-support dietary supplement products or different cat’s claw extracts) were placed on PTFE printed slides (EMS Sciences) and allowed to dry at room temperature overnight. Next, 10 ml of Thioflavin S (0.5% Fisher Scientific) solution was pipetted onto the dried samples in the wells and reacted for one minute. Then 10 ml of Thioflavin S solution was gently removed from the sample wells. Lastly, 3 ml of Vector Mount (Vector Labs) was added to the wells and cover slipped with 5 mm Phototech glass coverslips. The samples were viewed under fluorescent light and a prominent green fluorescence was indicative of Aβ fibrils or tau tangles present. A reduction and loss of the green fluorescence was indicative of disaggregation /reduction of Aβ fibrils or tau tangles.

### Circular dichroism (CD) spectroscopy

Circular dichroism (CD) spectroscopy evaluated the effects of percepta on disaggregation/reduction of β-sheet secondary structure on Aβ amyloid and tau protein fibrils/filaments. Many of these studies were previously reported^5^. In studies demonstrated here, 50 µM of tau-441 was induced with heparin (for 3 days at 37 °C) in the presence of percepta to determine whether percepta has the ability to halt tau tangle β-sheet secondary folding. CD spectra were collected at 25^o^ C on an AVIV CD Spectrometer 62DS. Measurements were carried out in a 0.5 mm path length quartz cuvette, over the range of 190–260 nm. The instrument was calibrated with an aqueous solution of (+)-10-camphorsulfonic acid. CD spectra consisted of an average of a series of scans made at 0.5 nm intervals.

### Negative stain electron microscopy

For negative stain electron microscopy (EM), 50 µM Aβ 1–40, 1–42 fibrils, or tau protein tangles (induced by heparin)^5,26,62–64^ were incubated at 37 °C in the presence or absence of memory-support dietary supplement products or cat’s claw extracts, at increasing wt/wt ratios. Aliquots were taken at 1, 3 and 7 days usually for analysis. For disruption of tau tangle studies, earlier time points (15 min, 1 h, 24, 48 and 72 h) were also taken for analysis to see how fast percepta was working. Negatively-stained Aβ amyloid and tau protein fibrils/filaments were prepared by floating piloform, carbon-coated grids on peptide solutions. After the grids were blotted and air-dried, the samples were stained with 2% (w/v) uranyl acetate and visualized with a JOEL 1400 transmission electron microscopy and digitally imaged using a Gatan digital camera and software.

### Statistical analysis

For all fibrillogenesis studies utilizing Thioflavin T fluorometry, comparisons of Aβ fibrils or tau protein tangles, in the presence or absence of test compounds (i.e. memory-support dietary supplements) or cat’s claw extracts were based on paired Student’s t tests with data shown as mean ± SEM. Significance was reported at the 95% (p < 0.05; *), 99% (p < 0.01; **) and 99.999% (p < 0.001; ***) confidence levels.

### Ranking order of dietary supplements/Cat’s claw products

Memory-support dietary supplements and cat’s claw extracts/products were ranked in order of efficacy for reduction of Aβ 1–42 fibrils and/or tau protein tangles using paired Student’s t tests and one-way ANOVA. Differences in ranking order included >>> statistically greater than 20%, >> statistically greater than 10%, and > statistically greater than 5%. An equal (=) sign indicated no significant difference between two or more products/extracts.
